# A Natural Polymer Captor for Immobilizing Polysulfide/Polyselenide in Working Li–SeS_2_ Batteries

**DOI:** 10.1007/s40820-021-00629-z

**Published:** 2021-04-05

**Authors:** Yin Zhang, Menglei Wang, Yi Guo, Lingzhi Huang, Boya Wang, Yunhong Wei, Peng Jing, Yueying Zhang, Yun Zhang, Qian Wang, Jingyu Sun, Hao Wu

**Affiliations:** 1 Engineering Research Center of Alternative Energy Materials and Devices of Ministry of Education, College of Materials Science and Engineering, Sichuan University, Chengdu, 610064 People’s Republic of China; 2 Key Laboratory of Advanced Carbon Materials and Wearable Energy Technologies of Jiangsu Province, College of Energy, Soochow Institute for Energy and Materials Innovations (SIEMIS), Soochow University, Suzhou, 215006 People’s Republic of China

**Keywords:** Li**–**SeS_2_ batteries, *Nicandra physaloides* pectin, Interlayer, Shuttle effect, Pouch cells

## Abstract

SeS_2_ has become a promising cathode material owing to its enhanced electrical conductivity over sulfur and higher theoretical specific capacity than selenium; however, the working Li**–**SeS_2_ batteries have to face the practical challenges from the severe shuttling of soluble dual intermediates of polysulfide and polyselenide, especially in high-SeS_2_-loading cathodes. Herein, a natural organic polymer, *Nicandra physaloides* pectin (NPP), is proposed to serve as an effective polysulfide/polyselenide captor to address the shuttling issues. Informed by theoretical calculations, NPP is competent to provide a Lewis base-based strong binding interaction with polysulfides/polyselenides via forming lithium bonds, and it can be homogeneously deposited onto a three-dimensional double-carbon conductive scaffold to finally constitute a polysulfide/polyselenide-immobilizing interlayer. *Operando* spectroscopy analysis validates the enhanced polysulfide/polyselenide trapping and high conversion efficiency on the constructed interlayer, hence bestowing the Li**–**SeS_2_ cells with ultrahigh rate capability (448 mAh g^−1^ at 10 A g^−1^), durable cycling lifespan (≈ 0.037% capacity attenuation rate per cycle), and high areal capacity (> 6.5 mAh cm^−2^) at high SeS_2_ loading of 15.4 mg cm^−2^. Importantly, pouch cells assembled with this interlayer exhibit excellent flexibility, decent rate capability with relatively low electrolyte-to-capacity ratio, and stable cycling life even under a low electrolyte condition, promising a low-cost, viable design protocol toward practical Li**–**SeS_2_ batteries.

**Supplementary Information:**

The online version contains supplementary material available at 10.1007/s40820-021-00629-z.

## Highlights

- Theoretical calculations reveal that *Nicandra physaloides* pectin can serve as Lewis base to realize strong interaction toward both lithium polysulfide and polyselenides.
- *Nicandra physaloides* pectin is introduced into a conductive double-carbon self-supporting film to build an anchoring and regulating interlayer for Li–SeS_2_ batteries.
- The Li–SeS_2_ pouch cells assembled with the interlayers deliver good flexibility and stability to demonstrate a viable strategy for developing working Li–SeS_2_ batteries.

**Supplementary Information:**

The online version contains supplementary material available at 10.1007/s40820-021-00629-z.

## Graphical abstract

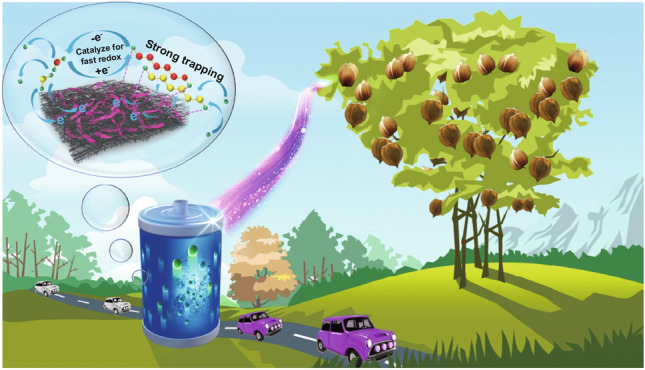

## Introduction

The fast growth of compact electronic devices and electric vehicles pushes researchers to focus on the exploitation of high energy density storage systems. Because of the high theoretical specific capacity (1675 mAh g^−1^) and energy density (2600 Wh kg^−1^), lithium-sulfur (Li–S) batteries have grabbed ever-increasing attention [1]. Nevertheless, the poor conductivity of sulfur, the shuttling of dissolved lithium polysulfides (Li_2_S_n_, 4 ≤ *n* ≤ 8), and volume expansion during discharge–charge process seriously hinder their practical applications [2]. Given this, lithium–selenium (Li–Se) batteries come into researchers’ view due to the higher conductivity of selenium (1 × 10^–3^ S m^−1^) than sulfur (5 × 10^–28^ S m^−1^) and equivalent volume specific capacity (3254 mAh cm^−3^) compared to Li–S batteries (3467 mAh cm^−3^) [3]. Nonetheless, the high industrial cost and low mass specific capacity (675 mAh g^−1^) of element selenium undermine its appeal as the electrode materials [4, 5].

To combine the advantages of S and Se, the solid solution selenium sulfide (SeS_2_) has become a promising cathode material [6]. Thanks to the higher specific capacity (1345 mAh g^−1^) than Se and the enhanced conductivity compared with S, the SeS_2_-based cathode materials have drawn great attention [7, 8]. In Amine’s pioneer work, SeS_2_, as a cathode material, exhibits tremendous potential for Li and Na storage [6]. Since then, the mechanism of electrochemical reaction and the cause of capacity degradation in ether-based electrolyte have been further researched. As the cathode material of Li metal batteries, SeS_2_ displays analogical electrochemical reaction to S and Se, meaning that SeS_2_ as the cathode also easily suffers the common shuttling problem of soluble dual intermediates of lithium polysulfides and lithium polyselenides (Li_2_S_n_ and Li_2_Se_n_, 4 ≤ n ≤ 8), which results in the irreparable loss of active species, fast capacity decay, and terrible self-discharge [9, 10]. Vast attempts have been implemented to inhibit the shuttling of soluble Li_2_S_n_/Li_2_Se_n_ and extend the lifespan of Li–SeS_2_ batteries. To date, carbon materials (*e.g.*, mesoporous carbon [11], double-layered hollow carbon sphere [12, 13], and CMK3 [14]) and polar materials (*e.g.*, metal nitrides [15–17] and metal sulfides [18, 19]) have been applied in fabricating SeS_2_ cathodes in order to physically or chemically capture active species. Dismayingly, most of these porous carbon materials only render limited absorption to dissolved Li_2_S_n_/Li_2_Se_n_, meanwhile the electrochemically inert carbon generally decreases the final SeS_2_ areal loading (< 2.0 mg cm^−2^) and actual SeS_2_ content (< 60 wt%) of electrode. Besides, both the complicated process and the high cost of the porous carbon materials still result in a long distance from the commercial application standards.

Recently, an increasing interest has focused on the novel adsorbents with merits of wide raw material sources, low price, and easy construction. Some organics with hydroxyl groups and amine groups have showed strong affinity with Li_2_S_n_ in Li–S batteries, so as to decrease the loss of active materials and meliorate the cycling performance [20]. For example, Dong and his co-workers have demonstrated that a polypropylene separator modified by tertiary amine layer can selectively bond with the soluble long-chain Li_2_S_n_ based on the classic “soft and hard acid–base (SHAB) theory” [21]. In addition, some natural organics, such as *β*-cyclodextrin polymer [22], lignin fibers [23], Gum Arabic [24], and proteins [25, 26], are combined with conductive materials to retard the dissolution and shuttling of Li_2_S_n_ for enhancing the electrochemical performance of Li–S batteries. Nevertheless, for all we know, there is no report referring to exploring natural organics to be simultaneously served as Li_2_S_n_ and Li_2_Se_n_ adsorbents in Li– SeS_2_ batteries, and the resulting effects to immobilizing these soluble intermediates and regulating their electrochemical performance are unknown. Moreover, very few attempts have been made so far on the fabrication of high-performance working Li– SeS_2_ batteries under realistic conditions, such as pouch cells with high SeS_2_ loading and lean electrolyte, yet which is a prerequisite for realizing the commercialization of Li–SeS_2_ batteries.

Herein, guided by density functional theory (DFT) calculations, we report that an effective natural organic polymer of *Nicandra physaloides* pectin (denoted as NPP) is competent to realize strong chemical interaction and immobilization toward both Li_2_S_n_ and Li_2_Se_n_, as it can ideally serve on the Lewis base to provide electrons and generate strong lithium bonds with the soluble dual intermediates. By introducing the NPP, assembled as a ribbon-like network, into a three-dimensional (3D) double-carbon conductive skeleton consisted of cotton carbon fibers (CF) and carbon nanotubes (CNTs), a freestanding hybrid film (CF@CNTs-NPP) is successfully constructed via a simple solution deposition method. The as-built CF@CNTs-NPP hybrid film with a 3D open structure not only favors the electrolyte infiltration without detaining too much electrolyte, but also functions as upper current collectors to ensure rapid ionic/electronic transport and good compatibility with high-areal-loading SeS_2_ cathodes. *Operando* analysis by in situ Raman spectroscopy further demonstrates the enhancement of trapping ability and conversion efficiency of Li_2_S_n_/Li_2_Se_n_ on the CF@CNTs-NPP film. With this designed interlayer, we greatly enhance the electrochemical performances of a easily made SeS_2_/CNTs cathode (70 wt% SeS_2_ content), as reflected by a considerably restrained self-discharge behavior, an outstanding cyclability with a capacity retention as high as 98.9% over 250 cycles at 1 A g^−1^ and 70% over 800 cycles at 2 A g^−1^, as well as an superhigh rate capability of 448 mAh g^−1^ at 10 A g^−1^, being the highest rate performance ever reported for Li–SeS_2_ batteries. Even at a largely increased SeS_2_ areal loading of 15.4 mg cm^−2^, it also achieves a high areal capacity over 6.6 mAh cm^−2^, which surpasses the traditional lithium-ion batteries. More significantly, the designed CF@CNTs-NPP interlayer gives rise to superior performances in a Li–SeS_2_ pouch cell configuration in terms of favorable flexibility, high discharge capacity (751 mAh g^−1^), low electrolyte-to-capacity ratio (≈7.8 µL (mA h)^−1^), high SeS_2_ utilization efficiency (> 55%), and stable cycling life (≈90.1% capacity retention over 70 cycles) even under high SeS_2_ loading and low electrolyte operation.

## Experimental Section

### Preparation of NPP

100 g commercial *Nicandra physaloides* (L.) Gaertn seeds were added into 1000 mL deionized water and kept stirring for an hour. Then, the mixture was filtered with gauze to obtain light yellow viscous filtrate. After the filtrate was frozen, the *Nicandra physaloides* pectin (NPP) were obtained by vacuum freeze-drying.

### Preparation of CF@CNTs-NPP, CF@CNTs, and CF Interlayers

The household cotton tissues (CT) composed by natural cotton were washing by deionized water and absolute alcohol, followed by dried at 60 °C for 12 h. Next, the carbon nanotubes (CNTs) dispersion with a content of 10 wt% purchased from Chengdu Organic Chemicals Co., LTD. Chinese Academy of Sciences was diluted with 100 mL deionized water to get a slurry of CNTs with a concentration of about 2 mg mL^−1^ after an ultrasonic treatment for 1 h. Then, the dried CT were immersed into the dispersion of CNTs with ultrasonic treatment for 30 min, followed by dried at 100 °C for 12 h. After that, the CT adsorbed CNTs were carbonized at 900 °C for 2 h with the Ar atmosphere to obtain CF@CNTs. The as-obtained films were cut into pieces with a diameter of 15 mm as the CF@CNTs interlayers. The NPP was dissolved into the mixture of deionized water and absolute alcohol (v/v = 4:1) with a content of 4 mg mL^−1^. Then, 60 μL NPP solution was dropped onto each CF@CNTs interlayer, followed by immersion in liquid nitrogen. After completely frozen, the solids were freeze-dried for 36 h and then dried at 60 °C for 12 h in a vacuum oven to obtain CF@CNTs-NPP interlayers. In addition, the CF@CNTs-NPP interlayers with the size of 2.5 × 4.5 cm^2^ for pouch cells are obtained through the same process except for adding 382 μL NPP solution. Besides, the CF interlayers were got by the direct carbonization of CT.

### Material Characterization

Field-emission scanning electron microscopy (FESEM) images were collected on Hitachi S-4800 instrument, which is equipped with energy-dispersive X-ray (EDX) system for elemental mapping. X-ray diffraction (XRD) measurement was taken on a Bruker DX-1000 diffractometer using Cu Kα radiation. The chemical element composition on the surface of composites was identified by X-ray photoelectron spectroscopy (XPS, AXIS Ultra DLD, Kratos) analysis. Fourier transform infrared (FT-IR) spectrum was performed on the Nicolet-5700 in the range of 400–4000 cm^−1^. Thermogravimetric analysis (TGA) was recorded on a simultaneous TGA/DSC-2 instrument (METTLER TOLEDO, USA). Raman spectra were collected on a Raman spectrophotometer (HORIBA Jobin Yvon, HR800, France) with 532 nm laser radiation. The UV–Vis data were collected by Shimadzu UV 3600. The square resistances were measured at room temperature using a ST-2258A digital four point probe test system (Suzhou Jingge Electronic Co., Ltd).

### Electrochemical Measurements

The selenium disulfides/carbon nanotubes (SeS_2_/CNTs) composite was prepared by mixing the commercial CNTs powder and SeS_2_ with a ratio of 70:30 by grinding, and the precursor was sealed in a reactor and heated at 160 °C for 10 h. The SeS_2_/CNTs composite (90 wt%) was mixed with sodium carboxymethyl cellulose (CMC, 5 wt%) and styrene butadiene rubber (SBR, 5 wt%) in deionized water solvent to form a slurry and cast onto carbon-coated aluminum foils. After dried in a vacuum oven at 50 °C for 24 h, the electrodes were cut into circular pieces with a diameter of 12 mm with the areal SeS_2_ loading about 1.3 mg cm^−2^. Besides, the slurry was coated onto carbon paper for higher SeS_2_ loadings from 4.5 to 15.4 mg cm^−2^. The coin cells (CR2032) were assembled in an argon-filled glove box for electrochemical tests using Li foil as anodes, Celgard 2400 as separators and Li–SeS_2_ electrolyte of 1 M lithium bis(trifluoromethanesulfonyl)-imide (LiTFSI) in 1,3-dioxolane (DOL) and 1,2-dimethoxyethane (DME) with volume ratio 1:1 and 2 wt.% LiNO_3_ as additive. The interlayers were placed between SeS_2_/CNTs cathodes and separators. For the soft-packed Li-SeS_2_ batteries, the SeS_2_/CNTs cathodes were cut into 2 × 4 cm^2^ pieces. Galvanostatic charge/discharge tests were performed using a multichannel battery test system (Neware CT-3008 W, China) within the potential range 1.7–2.8 V (*vs.* Li/Li^+^) at different current densities. Cyclic voltammetry (CV) measurements were taken using a PARSTAT multichannel electrochemical workstation (Princeton Applied Research, USA). Electrochemical impedance spectroscopy (EIS) was conducted over a frequency range of 100 kHz to 0.1 Hz with an applied amplitude of 5 mV.

## Results and Discussion

**Fig. 1:**
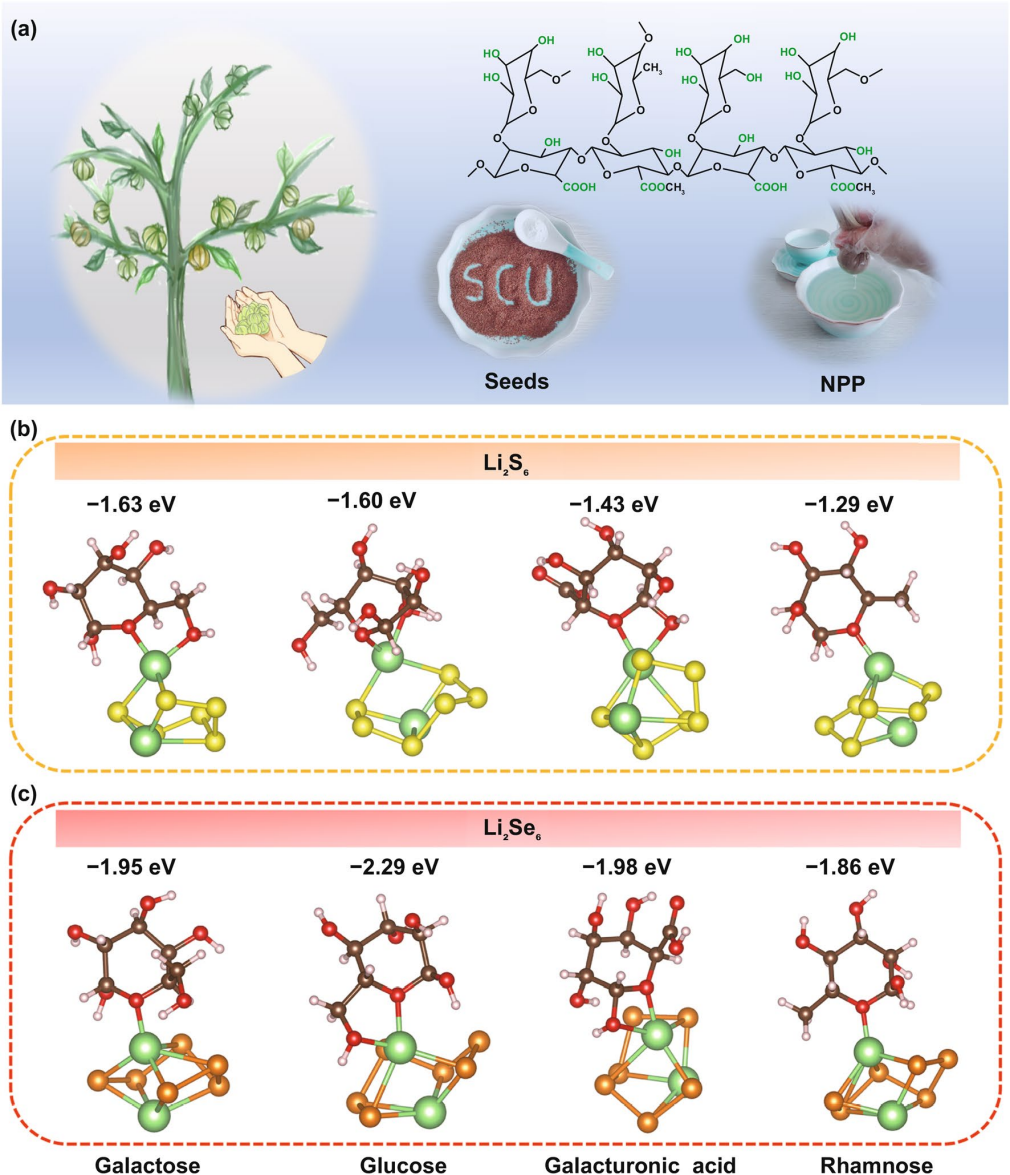
**a** Schematic diagram of the extraction process of the NPP. The optimized structures of four typical main components of NPP adsorbed with, **b** Li_2_S_6_ and **c** Li_2_Se_6_. The selenium, sulfur, oxygen, lithium, carbon and hydrogen are distinguished with orange, yellow, red, green, brown, and white. (Color figure online)

NPP, one of the most abundant and renewable biomass in nature, mainly comes from the natural *Nicandra physaloides* (L.) Gaertn, an annual herb of the Solanaceae family, which is native to Peru and widely distributed in Yunnan, Sichuan, Guizhou, and some other places in China [27, 28]. There is a layer of non-toxic, colorless, tasteless, edible colloids outside the seeds of the *N. physaloides*, and these colloids (*i.e.,* NPP) can be readily isolated by simple water extraction with great yields [28]. As shown in Fig. 1a, NPP is composed largely of a backbone of a *α*-1,4-linked galacturonic acid, many of which are esterified with methyl alcohol at the carboxylic acid, interspersed with a few rhamnose, galactose, and glucose residues linked to neutral side chains [29, 30]. To identify the chemical structure of NPP molecules, X-ray photoelectron spectroscopy (XPS) and Fourier transform infrared (FT-IR) spectrum were conducted. As shown in Fig. S1a, the XPS survey spectrum shows two strong binding energy peaks related to the C 1 s and O 1 s, respectively. The C 1 s spectrum of NPP (Fig. S1b) presents the existence of the carboxylate C (HO-C = O), ester C (O–C=O), hydroxyl C (C–OH), and non-oxygenated ring C (C–C/C=C) [31, 32]. Simultaneously, the O 1 s spectrum of NPP can be divide into two peaks, associated with the C = O and C–O–H/C–O–C bonds, respectively (Fig. S1c) [26, 33]. As for the FT-IR spectra of NPP (Fig. S2), the distinct peaks at 3419 and 2934 cm^−1^ belong to the stretching vibration of O–H and C–H bonds, respectively [34]. The peaks at 1728 and 1608 cm^−1^ are present due to the stretching vibrations of C=O bonds in ester and carboxyl, respectively [29, 35]. The peak at 1420 cm^−1^ are existing for the symmetric vibrations of O–C–O bonds, while the peak at 1238 cm^−1^ is attributed to the C–O–H deformation of pyranose rings [36]. The peaks located between 1200 and 1000 cm^−1^ correspond to the bending vibration of C–O–C glycosidic bonds in polysaccharide [37, 38]. Note that the weak absorption peaks in the low frequency region of 900–800 cm^−1^ are probably related to α-glycosidic bonds, which implies the existence of *α*-glycosidic bonds in NPP [34]. These results demonstrate that the NPP certainly is abundant in oxygen-containing groups, which could afford a powerful adsorption toward Li_2_S_n_/Li_2_Se_n_ and thus retard the shuttle effect.

To investigate the chemical interactions between the NPP and the Li_2_S_n_/Li_2_Se_n_, DFT calculations were firstly conducted by adopting Li_2_S_6_ and Li_2_Se_6_ as the models. The interaction between the key components of NPP (galactose, glucose, galacturonic acid, and rhamnose) and the Li_2_S_6_/Li_2_Se_6_ are displayed in Fig. 1b, c. As shown in Fig. 1b, because Li_2_S_n_ molecules have “electron cloud holes” to accept electrons from other molecules, the electron-rich functional groups in NPP, such as hydroxyl, ester, and epoxy groups, can provide electrons and form strong lithium bonds with Li_2_S_n_ according to the Lewis base-based chemical bonding, which is consist with the concept proposed by Zhang et al. [24, 39, 40]. Meanwhile, owing to the similar chemical property between Li_2_S_n_ and Li_2_Se_n_, the similar lithium bonds with Li_2_Se_n_ also exist toward NPP (Fig. 1c). Differential charge density analyses are displayed in Fig. S3, in which the increased electron densities between the lithium in Li_2_S_n_/Li_2_Se_n_ and the oxygen in NPP ascertain the formation of lithium bonds. Accordingly, the results of calculated adsorption energy are also represented in Fig. 1b, c. The constitutional units of NPP, such as galactose, glucose, galacturonic acid, and rhamnose, can afford the binding energies of −1.63, −1.60, −1.43, and −1.29 eV with Li_2_S_6_, respectively. Surprisingly, one can see that there are higher binding energies of −1.95, −2.29, −1.98, and −1.86 eV between galactose, glucose, galacturonic acid, and rhamnose with Li_2_Se_6_, respectively, as compared with Li_2_S_6_. Informed by these DFT results, it is predicted that the NPP not only possesses strong chemisorption to trap Li_2_S_n_, but also exhibits superior trapping and confinement to Li_2_Se_n_, implying that NPP can be used in Li–SeS_2_ batteries to retard the shuttling of Li_2_S_n_/Li_2_Se_n_.

**Fig. 2:**
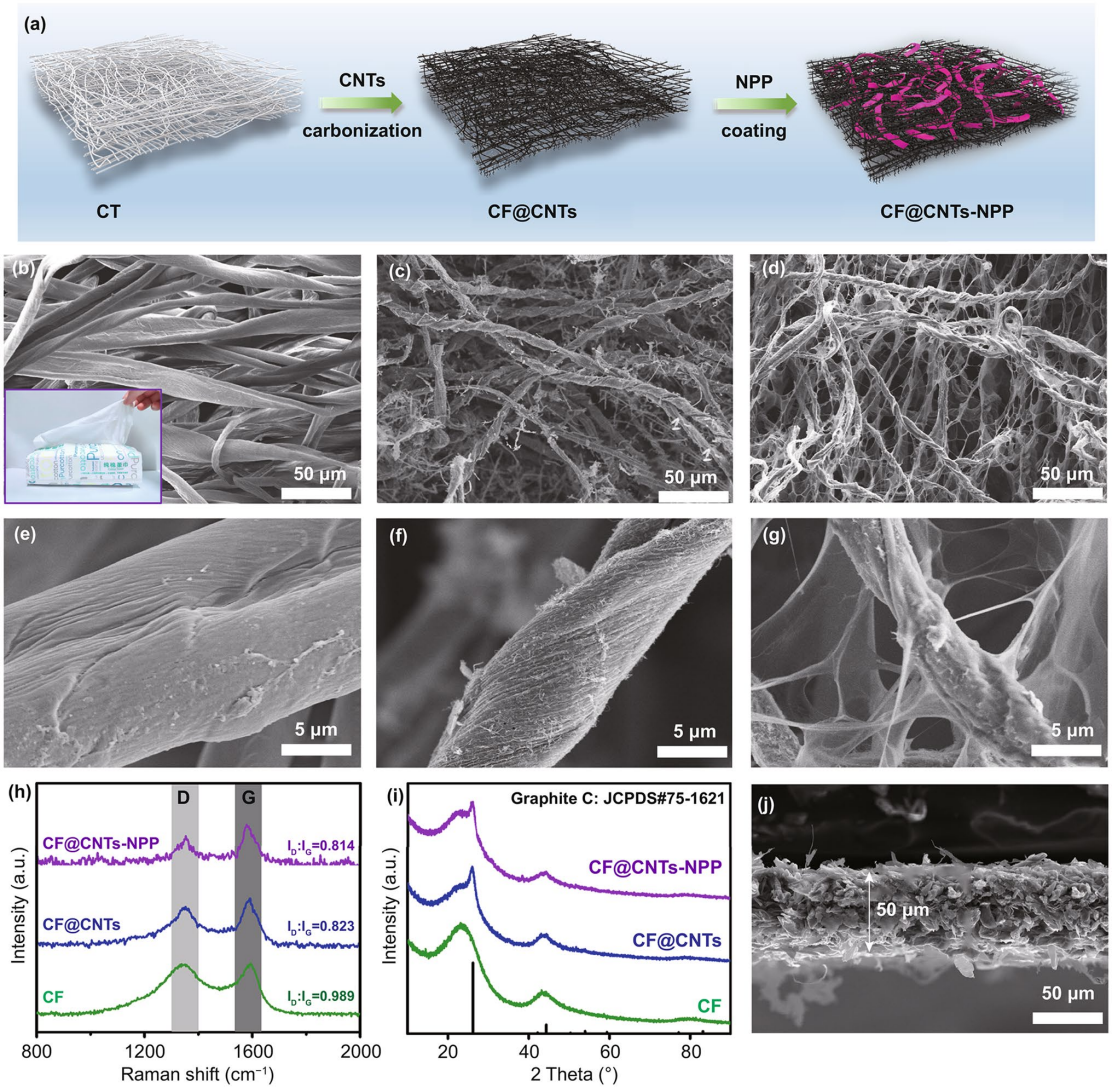
**a** Schematic diagram of the fabrication process of the CF@CNTs-NPP composite. Top-view FESEM images of **b**, **e** CF, **c**, **f** CF@CNTs, and **d**, **g** CF@CNTs-NPP composite. **h** Raman spectra and **i** XRD patterns of CF, CF@CNTs, and CF@CNTs-NPP. **j** Cross-sectional FESEM image of the CF@CNTs-NPP interlayer

Compared with complex architecture design of cathode materials, engineering functional interlayers is a cost-effective and easily producible strategy. As shown in Fig. 2a, the NPP was coated on the double-carbon CF@CNTs conductive frameworks to obtain CF@CNTs-NPP hybrid. The detailed preparation processes are depicted in the experimental section. The inexpensive household cotton tissues (denoted as CT, the insert of Fig. 2b), as the 3D carbon network precursor were immersed into and absorbed the suspension solution of CNTs. As revealed in Fig. 2b, e, the CT network possesses ample interspace constructed by cotton fibers with a diameter of ≈20 µm. Benefiting from the strong wettability of natural cotton [41], the CT can absorb the CNTs suspended in the aqueous solution to form CT@CNTs composite, as observed in their optical images in Fig. S4a, b. After that, the dried CT@CNTs composite was carbonized at 900 °C in an Ar atmosphere, in which the cotton fibers converted to CF with a shrinking size, as exhibited in Fig. S4c. From the further observation of field-emission scanning electron microscopy (FESEM) images in Fig. 2c, f, the intertwined CNTs not only cover the CF uniformly, but also fill in the interspace between CF, which constructs the self-supporting 3D double-carbon conductive CF@CNTs scaffold. By contrast, the CF obtained from direct carbonization of CT possesses 3D network constructed by carbon nanofibers with a smooth surface (Fig. S5). Since the introduction of CNTs, the as-built CF@CNTs has a higher graphitization degree than the bare CF, which can be testified by Raman spectroscopy. As depicted in Fig. 2h, the CF@CNTs displays a lower peak intensity ratio of *I*_D_/*I*_G_ (0.823) than that of bare CF (0.989) [42]. Moreover, the X-ray diffraction (XRD) pattern of CF@CNTs appears a sharp characteristic peak of graphitized carbon at 26.2° (Fig. 2i), while the bare CF only displays a broad peak of amorphous carbon around 26°. All these results indicate the more enhanced electrical conductivity of the CF@CNTs over the bare CF, which is further testified by square resistance tests, as exhibited in Table S1. Besides, as shown in Fig. S6, the CF and CF@CNTs both exhibit excellent flexibility and foldability.

Subsequently, the as-built CF@CNTs conductive framework was coated by the NPP through dropwise adding the NPP-containing dispersion solution so as to form the CF@CNTs-NPP hybrid. As shown in Fig. 2d, g, the NPP assembled as a ribbon-like network not only wrap the surface of CF@CNTs skeleton, but also is interconnected to form a 3D CF@CNTs-NPP network with enhanced flexibility. Note that the introduction of insulating organics NPP has almost no significant effect on reducing the conductivity of the CF@CNTs-NPP, which can be verified by the Raman spectroscopy (Fig. 2h) and the XRD patterns (Fig. 2i). Specifically, the CF@CNTs-NPP exhibits an approximate peak intensity ratio of *I*_D_/*I*_G_ (0.814) compared with CF@CNTs (0.823) and remains the characteristic XRD peak at 26.2° associated with the graphitized carbon. In addition, the CF@CNTs-NPP hybrid film maintains glorious flexibility and foldability with a diameter length of 15 mm (Fig. S7), while the thickness of CF@CNTs-NPP hybrid film was estimated to be ≈ 50 μm in the cross-sectional FESEM image (Fig. 2j).

**Fig. 3:**
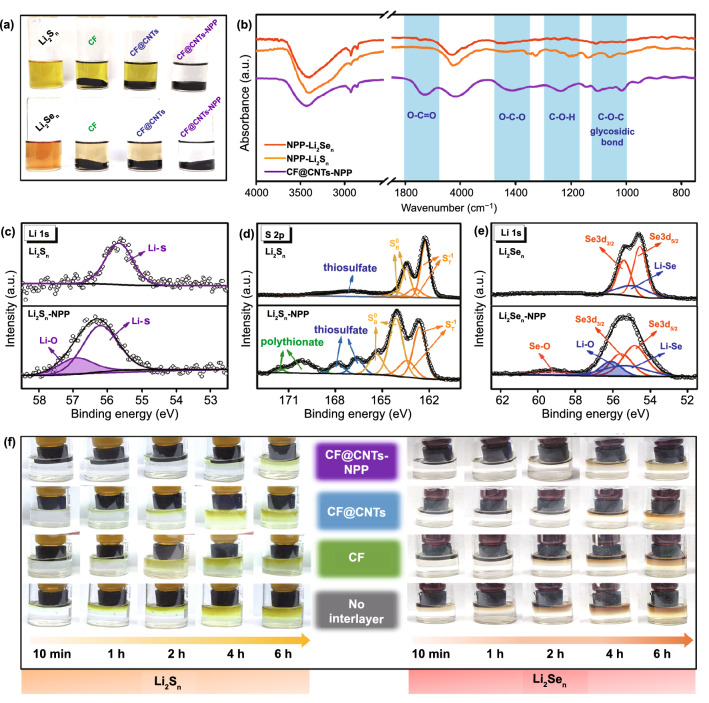
**a** Optical photographs of Li_2_S_n_ and Li_2_Se_n_ solutions contacted with CF, CF@CNTs, and CF@CNTs-NPP after 12 h. **b** FT-IR spectra of CF@CNTs-NPP, Li_2_S_n_-NPP, and Li_2_Se_n_-NPP. High-resolution XPS **c** Li 1s and **d** S 2p spectra of Li_2_S_n_ and Li_2_S_n_-NPP. **e** High-resolution XPS Li 1s spectra of Li_2_Se_n_ and Li_2_Se_n_-NPP. **f** Digital photographs of Li_2_S_n_ (left) and Li_2_Se_n_ (right) diffusion at different rest times

To verify the adsorption ability of the CF@CNTs-NPP hybrid, visualized Li_2_S_n_ and Li_2_Se_n_ adsorption experiments were carried out. As shown in Fig. 3a, the same amounts of CF, CF@CNTs, and CF@CNTs-NPP were added into the as-prepared Li_2_S_n_ and Li_2_Se_n_ solutions, respectively. After kept still for 12 h, all the solutions containing adsorbing materials become lighter than the bare one, especially for the Li_2_Se_n_ solution contacted with the CF@CNTs-NPP. Compared with the Li_2_S_n_ solution containing CF@CNTs-NPP, other Li_2_S_n_ solutions with adsorbents have no significant change. This result indicates the stronger confinement from the carbon matrix toward the Li_2_Se_n_ than Li_2_S_n_ [4, 43]. After adsorption experiments, the supernates were obtained and diluted for further UV–Vis absorption measurements. It can be observed from Fig. S8 that both the absorption intensity of Li_2_S_n_ and Li_2_Se_n_ solution after adding the CF@CNTs-NPP have a significant decline, confirming that the CF@CNTs-NPP hybrid is indeed capable of strong capturing both the soluble Li_2_S_n_ and Li_2_Se_n_ [44, 45].

The chemical interaction between S/Se species and CF@CNTs-NPP were excavated by FT-IR spectroscopy, which can unveil the change of functional groups in CF@CNTs-NPP hybrid before and after adsorption. As shown in Fig. 3b, the fresh CF@CNTs-NPP shows the main peaks that are almost identical to NPP. Interestingly, there are some obvious changes in the FT-IR spectra of CF@CNTs-NPP after Li_2_S_n_ adsorption (denoted as Li_2_S_n_-NPP) and after Li_2_Se_n_ adsorption (denoted as Li_2_Se_n_-NPP). For the Li_2_S_n_-NPP, all the peaks corresponding to C=O in ester, O–C–O, C-O–H, and C–O–C become weak distinctly, suggesting the chemical reactions occurred between the Li_2_S_n_ and the electron-rich oxygen-containing functional groups in CF@CNTs-NPP [46, 47]. In addition, these peaks in Li_2_Se_n_-NPP decrease more than those in Li_2_S_n_-NPP, confirming the stronger chemical interaction of NPP with Li_2_Se_n_ than Li_2_S_n_, which is consistent with the results of theoretical calculation.

To further explore the specific interactive relationship between Li_2_S_n_/Li_2_Se_n_ and CF@CNTs-NPP, the surface chemical properties of Li_2_S_n_, Li_2_Se_n_, Li_2_S_n_-NPP, and Li_2_Se_n_-NPP were further investigated by XPS. As presented in Fig. 3c, for the Li 1 s spectrum of the pristine Li_2_S_n_, the peak at 55.5 eV is assigned to the Li–S bond. After adsorption, the Li 1 s spectrum of Li_2_S_n_-NPP appears two peaks and undergoes a shift to a higher binding energy. The new peak located at 57.1 eV is associated with the formation of Li–O bond between the Li_2_S_n_ and the oxygen functional groups of NPP [48]. Meanwhile, the S 2p spectrum of Li_2_S_n_ shows two pairs of S doublets at 162 and 163 eV, corresponding to the terminal (S_T_^−1^) and bridge (S_B_^0^) S atoms, respectively (Fig. 3d) [49–51]. Besides, the formation of thiosulfate with a negligible intensity may be caused by the oxidation of sulfur atom during XPS detection process [52]. By comparison, the S peaks from Li_2_S_n_-NPP exhibit a considerable shift to a higher energy range, suggesting the reduction of electron cloud density along the sulfur chains [53, 54]. Moreover, the significant contribution between 166 and 172 eV is ascribed to thiosulfate and polythionate species formed by the redox reaction between the Li_2_S_n_ and the oxygen-containing functional groups in CF@CNTs-NPP [52, 55]. In Fig. 3e, the Se 3d spectrum displays two peaks at 55.4 and 56.3 eV attributed to the Se 3d_5/2_ and Se 3d_3/2_ states [56, 57], respectively, meanwhile the Li-Se bonds at 55.9 eV can be observed because of the overlapped region of Se 3d spectrum and Li 1s spectrum [58]. After adsorption, the appearance of Se–O and Li–O bonds verify the chemical interaction between the Li_2_Se_n_ and the oxygenic functional groups of NPP [58].

In order to visually demonstrate the distinctive capability of the CF@CNTs-NPP hybrid as an interlayer to capture both the Li_2_S_n_ and Li_2_Se_n_, the diffusion experiments are implemented by using the test device displayed in Fig. S9. Because of the concentration difference and the gravity effect, the Li_2_S_n_/Li_2_Se_n_ diffusion behavior can be observed by the color variation in the bottom of electrolyte. As shown in the left half of Fig. 3f, the tested bottle in the interlayer-free device exhibits a deep yellow color after 6 h, manifesting a high content of Li_2_S_n_ and thus a fast Li_2_S_n_ diffusion. Gratifyingly, the electrolyte in the device equipped with CF@CNTs-NPP interlayer only turns into pale yellow after 6 h, much lighter than that of other three samples, verifying the effective interception of CF@CNTs-NPP interlayer toward Li_2_S_n_. The similar results are embodied in Li_2_Se_n_ diffusion tests, as shown in right half of Fig. 3f. Obviously, the device assembled with CF@CNTs-NPP interlayer only has a slight color change even after 6 h. On the contrary, Li_2_Se_n_ diffuses quickly in the device without interlayer, and the colorless electrolyte at the bottom bottles turns into reddish brown after 6 h. All above diffusion tests reflect the effective adsorption of CF@CNTs-NPP interlayer, which can retard the diffusion and the shuttling of Li_2_Se_n_/Li_2_S_n_.

**Fig. 4:**
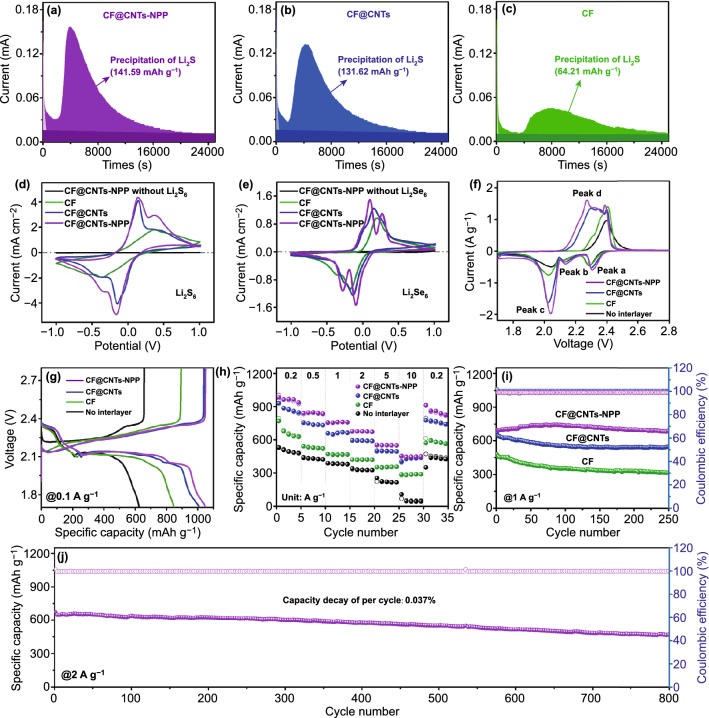
Potentiostatic discharge profiles of cells with **a** CF@CNTs-NPP, **b** CF@CNTs and **c** CF at 2.05 V. CV curves of symmetric cells with different electrodes **d** with or without 0.2 M Li_2_S_6_ and **e** with or without 0.2 M Li_2_Se_6_ at the scan rate of 1 mV s^−1^. **f** CV curves at the scan rate of 0.1 mV s^−1^. **g** Initial discharge–charge curves at 0.1 A g^−1^. **h** Rate capability. Cycling performance at **i** 1 A g^−1^ and **j** 2 A g^−1^ under room temperature

To examine if there exists a promoting effect of the CF@CNTs-NPP toward Li_2_S_n_ conversion, Li_2_S precipitation experiments were performed. As exhibited in Fig. 4a–c, the responsiveness of Li_2_S nucleation on CF@CNTs-NPP is earlier than that on CF@CNTs and CF, indicating the better catalytic promoting effect of CF@CNTs-NPP for Li_2_S_n_ transformation [59, 60]. Moreover, the CF@CNTs-NPP exhibits the highest precipitation current (0.16 mA) and conversion capacity of 141.59 mAh g^−1^ compared to the CF@CNTs (0.13 mA, 131.62 mAh g^−1^) and CF (0.05 mA, 64.21 mAh g^−1^). These results attest that the CF@CNTs-NPP can substantially reduce the overpotential of Li_2_S nucleation and accelerate the interfacial electron transfer kinetics for Li_2_S precipitation [61, 62]. To better identify the potential catalytic effect of the CF@CNTs-NPP on enhancing the conversion of Li_2_S_n_ and Li_2_Se_n_, symmetric cells were further assembled by adopting two selfsame electrodes with Li_2_S_6_ and Li_2_Se_6_ electrolyte, respectively. The experimental symmetric cell employs two CF@CNTs-NPP electrodes, while the other two control cells respectively contain two CF and CF@CNT electrodes. The cyclic voltammetry (CV) curves of these assembled symmetric cells were detected at a scan rate of 1 mV s^−1^. The CV curves of the symmetric cells with a Li_2_S_6_/Li_2_Se_6_-free electrolyte were measured, which verifies no contribution to the capacitive current, as shown in Fig. 4d, e. In the presence of the CF@CNTs-NPP, the cells either with Li_2_S_6_ (Fig. 4d) or with Li_2_Se_6_ (Fig. 4e) show the sharpest redox peaks together with the strongest current response when compared with the other control cells, implying the enhanced electrochemical reversibility along with favorable Li_2_S_n_ and Li_2_Se_n_ redox conversion [63, 64].

To exhibit the availability of CF@CNTs-NPP, the electrochemical performance of Li–SeS_2_ coin cells with the CF@CNTs-NPP as interlayers was tested using SeS_2_/CNTs composite cathode with a 70 wt% SeS_2_ content (Fig. S10). For comparison, the cells without interlayers and with CF and CF@CNTs interlayers were also assembled with the same composite cathodes as the contrast samples. The CV curves of the four types of cells were conducted at a scanning rate of 0.1 mV s^−1^ with the voltage range of 1.7–2.8 V. As shown in Fig. 4f, the cell with CF@CNTs-NPP interlayer exhibits three cathodic peaks, corresponding to the typical successive lithiation conversion processes from SeS_2_ to soluble long-chain Li_2_S_n_ and long-chain Li_2_Se_n_, then, to the final products of Li_2_S_2_/Li_2_S and Li_2_Se. The anodic peak is considered to the gradual delithiation of Li_2_S_2_/Li_2_S and Li_2_Se [14]. Compared to the cells without interlayer and with CF interlayer, the cells with CF@CNTs-NPP and CF@CNTs interlayers show much higher peak currents, higher reduction potentials, and lower oxidation potentials, indicating the rapid redox kinetics associated with the excellent conductivity of the CF@CNTs skeleton. This is further testified by the reduction of charge-transfer resistance of the cells with CF@CNTs-NPP and CF@CNTs interlayers due to the construction of CF@CNTs skeleton (Fig. S11) [65].

The initial discharge–charge curves of the four cells at 0.1 A g^−1^ are exhibited in Fig. 4g. Specifically, the cell with CF@CNTs-NPP interlayer shows four discharge plateaus at around 2.32, 2.17, 2.14, and 2.04 V, ascribed to the generation of soluble high-order Li_2_S_n_ and Li_2_Se_n_, then, the transformation to the final products of Li_2_S_2_/Li_2_S and Li_2_Se. The four similar discharge platforms appear in the curve of the cell with CF@CNTs interlayer. However, there are only three plateaus in the discharge curves of the cell with CF interlayer and the cell without interlayer, implying the severe polarization and high resistance caused by the poor conductivity and weak adsorption to Li_2_S_n_ and Li_2_Se_n_. Remarkably, the initial discharge and charge capacities of the cell with CF@CNTs-NPP interlayer are 1049 and 1050 mAh g^−1^, respectively, which are higher than the cells with CF@CNTs (1012/1039 mAh g^−1^), CF interlayer (850/894 mAh g^−1^) and without interlayer (625/657 mAh g^−1^), indicating that the relatively superior utilization of SeS_2_ can be achieved in the employment of CF@CNTs-NPP interlayer.

The enhanced redox kinetics of the cell with interlayer is further demonstrated by the excellent cycling response to continuous variation of current densities (Figs. 4h and S12). When cycled at 0.2, 0.5, 1, 2, 5, and 10 A g^−1^, the cell with CF@CNTs-NPP interlayer delivers the average discharge capacities of 964, 843, 758, 674, 551, and 448 mAh g^−1^, respectively, which is the highest capacity over 2 A g^−1^ in ever reported for Li-SeS_2_ batteries, as summarized in Fig. S13. As the current density successively turns back to 0.2 A g^−1^, the capacity can rapidly restore to 910 mAh g^−1^, demonstrating the excellent high-rate response and reversibility of the cell. Attributed to the high conductivity of CF@CNTs framework, the cell with CF@CNTs interlayer also exhibits good capacity retention from 880 to 427 mAh g^−1^ as the rate increases from 0.2 to 10 A g^−1^. Nevertheless, the capacity declines dramatically to 774 mAh g^−1^ when the current density reverts to 0.2 A g^−1^. In sharp contrast, the discharge capacity of the cell with CF interlayer fades sharply from 670 to 285 mAh g^−1^, while the cell without interlayer shows the worst capacity retention from 499 to 57 mAh g^−1^ when the rate increases from 0.2 to 10 A g^−1^. Apart from that, the comparison of cycle performance at a current density of 1 A g^−1^ is exhibited in Figs. 4i and S14. The cell without interlayer only exhibits an initial discharge capacity of 397 mAh g^−1^ with unstable and low Coulombic efficiency in the latter part of the cycle, as depicted in Fig. S14. As shown in Fig. 4i, after the introduction of the CF, CF@CNTs, and CF@CNTs-NPP interlayers, the initial discharge capacities increase to 480, 674, and 696 mAh g^−1^, respectively, but the rapid capacity decay happened in the cells with CF and CF@CNTs interlayers over 250 cycles. Noticeably, the cell with CF@CNTs-NPP interlayer can still remain a high capacity of 688 mAh g^−1^ after 250 cycles with corresponding capacity retention ratio as high as 98.9%, demonstrating its outstanding cycling stability. Furthermore, the long-term cycling test of the cell with CF@CNTs-NPP is displayed in Fig. 4j. It maintains the capacity of 470 mAh g^−1^ even after 800 cycles at 2 A g^−1^ together with a stable CE of above 99%, presenting a high capacity retention ratio of 70% and a low capacity decay of 0.037% per cycle. This indicates that the introduction of NPP into the hybrid interlayer is very effective to restrain the shuttling of polysulfide/polyselenide immediate and reduce the loss of active species for achieving a long lifespan in Li–SeS_2_ batteries.

**Fig. 5:**
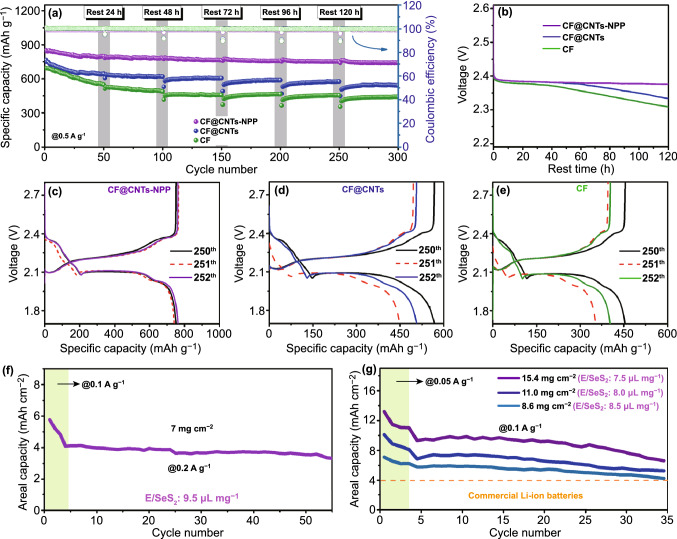
**a** Cycling stability at 0.5 A g^−1^ after different rest periods of 24, 48, 72, 96, and 120 h. **b** Open-circuit voltage curves of the cells during the 120 h rest. **c–e** Discharge–charge curves before and after 120 h rest. Cycling performance of the high-SeS_2_-loading cathodes of **f** 7 mg cm^−2^ and **g** 8.6, 11, and 15.4 mg cm^−2^ of the Li–SeS_2_ cells with the CF@CNTs-NPP interlayers

The serious self-discharge issue caused by the dissolution of intermediates into electrolyte is also a critical issue in Li-SeS_2_ batteries. Thus, we further tested the self-discharge behaviors of the cells with CF, CF@CNTs, and CF@CNTs-NPP interlayers by implementing intermittent discharge/charge tests with the rest time of 24 h after 50 cycles, 48 h after 100 cycles, 72 h after 150 cycles, 96 h after 200 cycles, and 120 h after 250 cycles. As shown in Fig. 5a, after the fitful discharge–charge tests of 300 cycles at 0.5 A g^−1^, the cell with CF@CNTs-NPP interlayer still keeps the capacity of 741 mAh g^−1^ and delivers a capacity retention of 87%. Contrarily, the capacity retentions of the cells with CF@CNTs and CF interlayers are only 74% and 61%, respectively. At the same time, the change of the open-circuit-voltages (OCV) during 120 h rest time after 250 cycles is displayed in Fig. 5b. The OCV of the cell with CF interlayer started to drop significantly after 20 h rest time and decline sharply to 2.3 V after 120 h. The drop in OCV of the cell with CF@CNTs interlayer is ameliorated, which started to drop noticeably after 60 h rest time and finally decline to 2.33 V. As for the cell with CF@CNTs-NPP interlayer, its OCV experiences only a very slow reduction process to 2.37 V. Even after resting for 120 h, the cell with CF@CNTs-NPP interlayer still remains a high discharge capacity of 741 mAh g^−1^ and a low self-discharge ratio of only 1.5% (Fig. 5c). In sharp contrast, the cells with CF@CNTs and CF interlayers exhibited high self-discharge ratio of 20.8% (Fig. 5d) and 21.9% (Fig. 5e), respectively. These results further manifest the effectivity of NPP in immobilizing soluble Li_2_S_n_/Li_2_Se_n_ to suppress the self-discharge in Li–SeS_2_ batteries.

The endurance under varying temperature atmosphere is of great importance for the practical application of batteries. Aiming to identify the thermal and frigid endurance performance, the cells with CF@CNTs-NPP interlayers were further tested at a raised temperature of 55 °C and a subzero temperature of −5 °C. As shown in Fig. S15a, c, when cycled at 0.2, 0.5, 1, 2, 5, and 10 A g^−1^, the cell is able to show the average discharge capacities of 903, 758, 661, 604, 551, and 474 mAh g^−1^ at 55 °C, while it also delivers the average discharge capacities of 611, 501, 397, 255, 142, and 77 mAh g^−1^ at −5 °C, respectively. Besides, under such extreme temperature condition, it still shows exceptionally good cycling performance both at 55 °C (474 mAh g^−1^ remaining after 250 cycles) and −5 °C (329 mAh g^−1^), as depicted in Fig. S15b and d. Nevertheless, the cells with the CF and CF@CNTs interlayers under raised and subzero temperatures both exhibit worse rate capability and cycle performance than the cell with CF@CNTs-NPP interlayer.

To meet the demand of high energy density Li–SeS_2_ batteries, a high SeS_2_ areal loading and a low electrolyte-to-selenium sulfide (E/SeS_2_) ratio are commonly essential. However, most of recent studies on Li–SeS_2_ batteries focused solely on the high capacity performance under a low SeS_2_ areal loading (< 2.0 mg cm^−2^) and a high E/SeS_2_ ratio (> 15 μL mg^−1^) [16, 66]. As such, we further fabricated a series of high-areal-loading and low-E/SeS_2_-ratio cells with the CF@CNTs-NPP interlayers. As shown in Fig. S16a, with a high areal SeS_2_ loading of 4.5 mg cm^−2^ and a relatively low E/SeS_2_ ratio of 10 µL mg^−1^, the resultant cathode shows the reversible discharge capacities of 793, 653, 544, and 499 mAh g^−1^ as the current density increase from 0.1 to 1 A g^−1^. In addition, the cycling tests also show that a high reversible capacity of 600 and 450 mAh g^−1^ can be retained after 100 cycles at 0.2 and 0.5 A g^−1^ with a good capacity retention of 82.3% and 89.3%, respectively (Fig. S16b). Moreover, by increasing the areal loading to 7.5 mg cm^−2^ along with a lower E/SeS_2_ ratio of 9.5 µL mg^−1^, the cell with CF@CNTs-NPP interlayer still delivers a high initial discharge capacity of 550 mAh g^−1^ at 0.2 A g^−1^, and it can keep a stable cycling over 50 cycles (Fig. 5f). More remarkably, even when the SeS_2_ loadings were dramatically increased to 8.6, 11.0, 15.4 mg cm^−2^ while further lowering the E/SeS_2_ ratio to 8.5, 8.0, and 7.5 µL mg^−1^, respectively, as shown in Fig. 5g, the cells are capable of achieving the areal capacities as high as 4.2, 5.3, and 6.6 mAh cm^−2^ after 30 cycles at 0.1 A g^−1^, which manifest strong competitiveness compared to the state-of-the-art lithium-ion batteries.

**Fig. 6:**
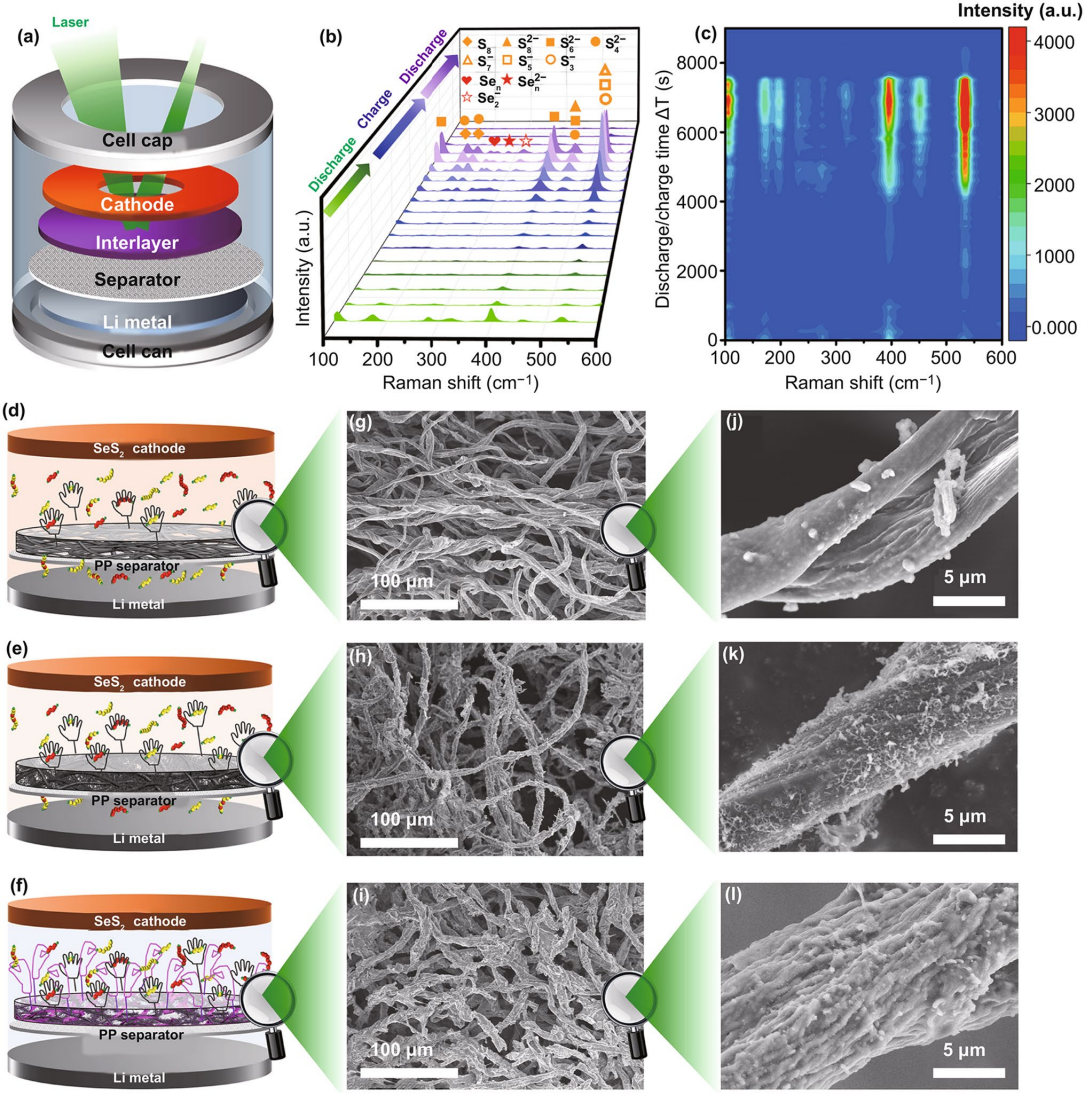
**a** Cell configuration for *Operando* Raman test; **b** *Operando* Raman spectra and **c** the corresponding contour plot at different discharge and charge states. Schematic illustration of the operation of the Li**–**SeS_2_ batteries with **d** CF, **e** CF@CNTs, **f** CF@CNTs-NPP interlayers and **g–l** the corresponding FESEM images of different interlayers toward the cathodes after 100 cycles

In order to gain an insight into the adsorption and conversion of CF@CNTs-NPP interlayer to Li_2_S_n_ and Li_2_Se_n_, *operando* characterization with in situ Raman spectroscopy was tested to track the existence and transformation of Li_2_S_n_ and Li_2_Se_n_ on the CF@CNTs-NPP interlayer during the overall redox process, and Fig. 6a presents the tested cell configuration. As shown in Fig. 6b, c, during the early stage of the first discharge process, it is clearly observed that the Li_2_S_n_ including Li_2_S_6_, Li_2_S_4_ and Li_2_S_8_ can be easily determined by their characteristic peaks located at 107, 398, and 453 cm^−1^ in the Raman spectra [67, 68]. Moreover, the active anionic free radicals of S_7_^−^, S_5_^−^, and S_3_^−^ centered at 533 cm^−1^ are observed [69]. Meanwhile, the characteristic peaks of chain-liked Se_n_^2−^ (260 cm^−1^) and active anionic free radical Se_2_^−^ (320–350 cm^−1^) are also observed [70, 71]. All above results indicate that the Li_2_S_n_ and Li_2_Se_n_ generated during the early stage of the first discharge process can be well anchored by the CF@CNTs-NPP interlayer. With the discharge process, the peaks of Li_2_S_n_ and Li_2_Se_n_ display a gradual disappearance, demonstrating that the Li_2_S_n_ and Li_2_Se_n_ have been converted to Li_2_S_2_/Li_2_S and Li_2_Se. During the following charge process, the Li_2_S_n_ and Li_2_Se_n_ signals regenerate along with new peaks corresponding to the S_8_ (170–200 cm^−1^) [69] and chain-like Se_n_ (230–260 cm^−1^) [58, 72], declaring that both the Li_2_S_n_ and Li_2_Se_n_ trapped on the CF@CNTs-NPP interlayer are able to experience a reversible electrochemical conversion. During the second discharge process, the peaks about Li_2_S_n_ and Li_2_Se_n_ become stronger than the first discharge process and gradually disappeared again, further confirming the powerful immobilization and conversion of CF@CNTs-NPP interlayer to Li_2_S_n_ and Li_2_Se_n_.

To further verify the correlation of the adsorption function and structural stability of interlayers with the electrochemical performances of Li–SeS_2_ batteries, a simplified model displaying the difference between the function of three kinds of interlayers in the immobilization process of Li_2_S_n_/Li_2_Se_n_ is illustrated in Fig. 6d-f. As shown in Fig. 6d, the soluble Li_2_S_n_/Li_2_Se_n_ can be restricted in the cathode region slightly because of the introduction of CF interlayer. When CNTs are incorporated into the CF interlayer, as shown in Fig. 6e, the CF@CNTs interlayer possesses more abundant physical adsorption sites and higher electric conductivity, which work as a physical barrier for confining Li_2_S_n_/Li_2_Se_n_ and a “vice-electrode” with fast electronic transport channel to realize the reuse of Li_2_S_n_/Li_2_Se_n_ [73]. As for CF@CNTs-NPP interlayer (Fig. 6f), it not only has the inherent functions from CF@CNTs scaffold, but also affords a strong chemisorption to immobilize Li_2_S_n_/Li_2_Se_n_ owing to the ample oxygenic functional groups of NPP, thus retarding the shuttle effect and prolonging the lifespan of Li–SeS_2_ batteries. This can be corroborated by the morphologies of three kinds of interlayers after 100 cycles in Fig. 6g–i. As shown in Fig. 6j–l, compared to the interlayers before cycles, all three kinds of interlayers still keep the interwoven fiber structure except the increase of surface roughness, which is attributed to the deposition of S/Se species. As clearly shown in Fig. 6l, the diameters of fibers in CF@CNTs-NPP interlayer enlarge markedly toward the cathode side, but it is not obvious toward the separator side (Fig. S17), testifying the efficient absorptivity of NPP to Li_2_S_n_/Li_2_Se_n_. In contrast, the fibers in CF (Fig. 6j) and CF@CNTs interlayer toward the cathode side (Fig. 6k) have nearly unchanged.

The element distribution of the CF@CNTs-NPP interlayer toward the cathode and separator was further inquired by energy-dispersive X-ray (EDX) system. Based on the elemental mapping results, as shown in Fig. S18, distinct sulfur and selenium signals with a homogeneous distribution can be clearly observed in the CF@CNTs-NPP interlayer toward the cathode side, while the signals of sulfur and selenium are relatively weaker toward the separator side. Besides, the component ratios of the three cycled interlayers are shown in Fig. S19. The CF@CNTs-NPP interlayer toward the cathode side possesses the highest sulfur and selenium mass content, suggesting that the CF@CNTs-NPP interlayer effectively blocks the dissolution and shuttling of soluble Li_2_S_n_/Li_2_Se_n_ so as to largely immobilize and confine them in the cathode side, which leads to reduced loss of the active species and enhanced lifespan of Li–SeS_2_ batteries.

In addition, the structural stability of lithium (Li) metal in the anode is also crucial to the electrochemical performances of Li–SeS_2_ batteries. To explore the relevance of the adsorption function of NPP with the structural stability of solid electrolyte interphase (SEI) and Li metal, the surface morphologies and element distribution of the Li metal in different kinds of Li-SeS_2_ cells after 10 cycles at 1 A g^−1^ were conducted by field-emission scanning electron microscopy with energy-dispersive X-ray (EDX) system. As shown in Fig. S20a-h, the surface of the Li metal in cell with CF@CNTs-NPP interlayer is relatively smooth and compact, while the cells with CF@CNTs and CF interlayers and the cell without interlayer all exhibit corrosive and scraggy surface of Li metal. Besides, the component ratios of the four kinds of cycled Li metal are shown in Fig. S20i-l. The Li metal of the cell with CF@CNTs-NPP interlayer possesses the lowest sulfur and almost undetectable selenium mass content. All above results suggest that the CF@CNTs-NPP interlayer can effectively block the dissolution and shuttling of soluble Li_2_S_n_/Li_2_Se_n_ so as to largely alleviate the depletion of LiNO_3_ and the corrosion of fresh Li, contributing to stable SEI and significantly improved lifespan of Li–SeS_2_ batteries under practical conditions [74–76].

**Fig. 7:**
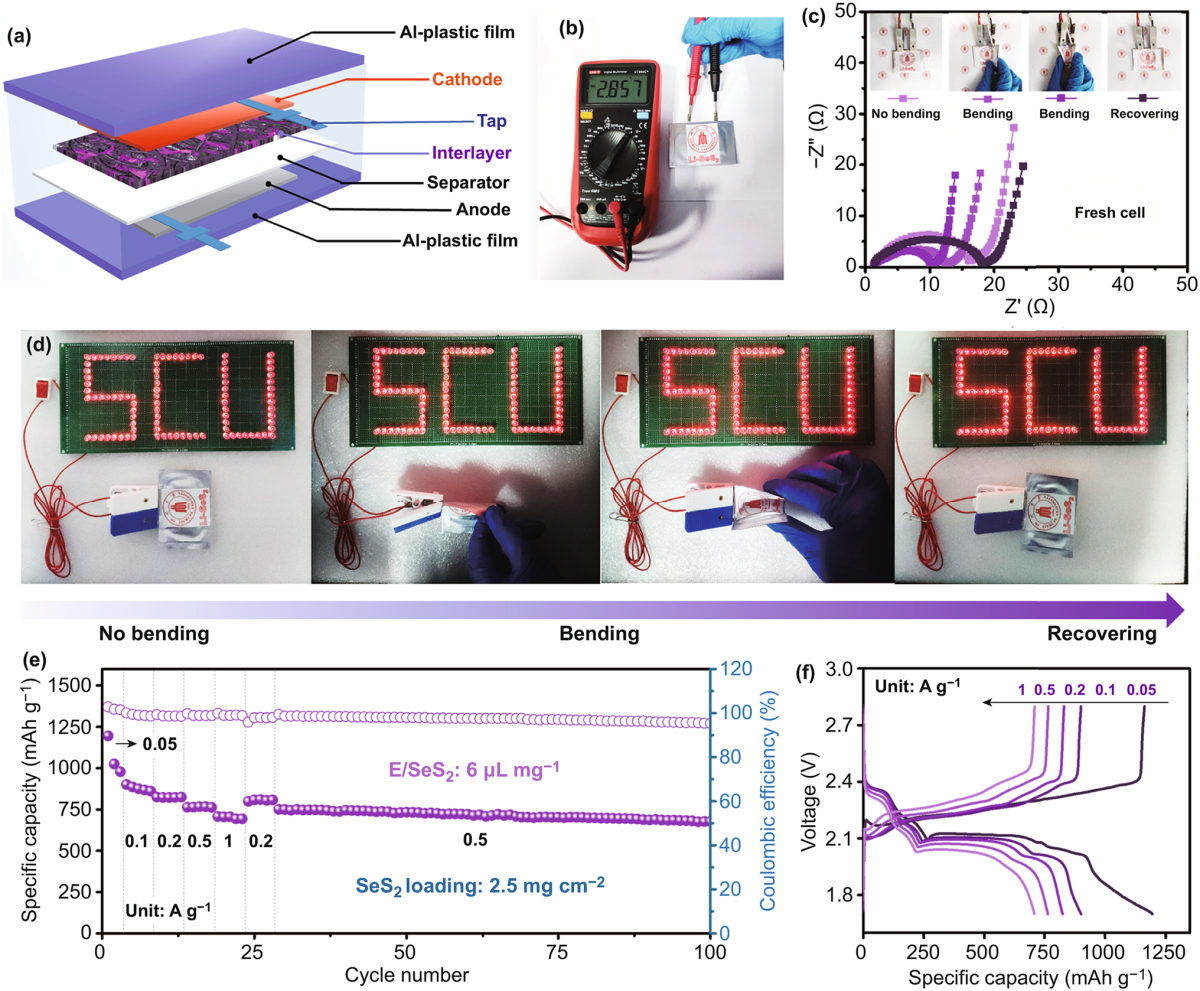
**a** Schematic diagram and **b** the open-circuit voltage of a Li**–**SeS_2_ pouch cell with the CF@CNTs-NPP interlayer. **c** Nyquist plots of the pouch cell before and after bending. **d** Demonstration of the pouch cell under various bending states to continuously light up the red light-emitting diodes (LED). **e** Electrochemical performance and **f** typical discharge–charge curves of the pouch cell at various current densities

The outstanding performance of the CF@CNTs-NPP interlayer in high-SeS_2_-loading and low-E/SeS_2_-ratio coin cells inspired us to further investigate the performance in pouch cells, which is a prerequisite for realizing practical application of Li–SeS_2_ batteries. As such, Li–SeS_2_ pouch cells with the CF@CNTs-NPP interlayers were readily assembled with a size of 2 × 4 cm^2^ and a typical SeS_2_ loading of 2.5 mg cm^−2^ with a much lower E/SeS_2_ ratio of 6 µL mg^−1^, as illustrated in Fig. 7a. In order to confirm the stability of this pouch cell configuration, the electrochemical impedance spectroscopy (EIS) curves under various bending states are given in Fig. 7c. Surprisingly, the charge-transfer resistance of the pouch cell is lower than that of the coin cell and keeps around 15 Ω, which is due to the intimate contact between the electrodes and electrolyte as well as the fast charge-transfer dynamics [77, 78]. Additionally, the Li–SeS_2_ pouch cell is able to yield an output voltage of 2.857 V (Fig. 7b) that can light up a “SCU” device containing 93 red light-emitting diodes (LEDs) under various bending states, indicating its outstanding flexibility and stability (Fig. 7d). Furthermore, as exhibited in Fig. 7e, f, the Li–SeS_2_ pouch cell exhibits a good rate capability with high discharge capacities of 1066, 879, 824, 764, and 701 mAh g^−1^ at 0.05, 0.1, 0.2, 0.5, and 1 A g^−1^, respectively, corresponding to low electrolyte-to-capacity ratio of ≈5.6, 6.8, 7.2, 7.8, and 8.5 µL (mAh)^−1^ and ≈79%, 65%, 61%, 57%, and 52% of SeS_2_ utilization efficiency. Meanwhile, the subsequent cycling test at 0.5 A g^−1^ shows that the pouch cell delivers a high discharge capacity of 751 mAh g^−1^ and retains a satisfying capacity retention of 90.1% after 70 cycles. Notably, this is corresponding to a relatively low electrolyte-to-capacity ratio of ≈7.8 µL (mA h)^−1^, indicating a decent utilization of SeS_2_ (over 55%) even under operation with a low E/SeS_2_ ratio (6 µL mg^−1^). This implies the great potential of CF@CNTs-NPP interlayers for practical applications in working Li–SeS_2_ batteries.

## Conclusions

In summary, under the guidance of theoretical calculations, we have developed a feasible and simple protocol to engineer a Li_2_S_n_/Li_2_Se_n_-regulating interlayer through depositing the natural NPP onto a 3D double-carbon conductive scaffold for high-performance Li–SeS_2_ batteries. Benefiting from the ample oxygenic functional groups, NPP can provide a potent chemical adsorption toward Li_2_S_n_/Li_2_Se_n_ based on the Lewis base chemical interaction and thereby effectively restrict the shuttling effect. Meanwhile, with the aid of the 3D open double-carbon conductive framework, the CF@CNTs-NPP as polysulfide/polyselenide shielding interlayer demonstrates effective promoting function to facilitate the Li_2_S_n_/Li_2_Se_n_ conversion, improve their redox kinetics, and enhance the SeS_2_ utilization efficiency. As a result, the cells assembled with the CF@CNTs-NPP interlayers show ultrahigh rate capability, durable cycling lifespan, and high areal capacity even at high SeS_2_ loading. More meaningfully, the pouch cells with high SeS_2_ loading and low E/SeS_2_ ratio have been successfully demonstrated to possess remarkable advantages in outstanding flexibility, high specific capacity, and stable cycling life. This work would manifest a prospective design and engineering protocol to develop low-cost, convenient, and feasible Li–SeS_2_ batteries for practical applications.

## Supplementary Information

Below is the link to the electronic supplementary material.

Supplementary file1 (PDF 2109 kb)
