# Supplementary material for: A Natural Polymer Captor for Immobilizing Polysulfide/Polyselenide in Working Li–SeS2 Batteries
Source: Nanomicro Lett. 2021 Apr 5;13:104. doi: 10.1007/s40820-021-00629-z (PMC8021686; doi:10.1007/s40820-021-00629-z)
Supplement: Supplementary file 1 — Supplementary file1 (PDF 2109 kb) [file 40820_2021_629_MOESM1_ESM.pdf]

Supporting Information for

A Natural Polymer Captor for Immobilizing Polysulfide/Polyselenide in Working Li–SeS_2 Batteries

Yin Zhang[1], Menglei Wang[2], Yi Guo[1], Lingzhi Huang[1], Boya Wang[1], Yunhong Wei[1], Peng Jing[1], Yueying Zhang[1], Yun Zhang[1,]*, Qian Wang[1], Jingyu Sun[2,]*, Hao Wu[1,]*

[1]Engineering Research Center of Alternative Energy Materials & Devices, Ministry of Education, College of Materials Science and Engineering, Sichuan University, Chengdu, 610064, P. R. China

[2]College of Energy, Soochow Institute for Energy and Materials Innovations (SIEMIS), Key Laboratory of Advanced Carbon Materials and Wearable Energy Technologies of Jiangsu Province, Soochow University, Suzhou, 215006, P. R. China

*Corresponding authors. E-mail: y_zhang@scu.edu.cn (Yun Zhang), sunjy86@suda.edu.cn (Jingyu Sun), hao.wu@scu.edu.cn (Hao Wu)

S1 Supporting Experimental Details

S1.1 Visualized Li_2S_n and Li_2Se_n Adsorption and Diffusion Test

Li_2S_n solution was prepared by dissolving S and Li_2S with a molar ratio of 1:5 in DOL/DME mixed solution with volume ratio 1:1, followed by magnetically stirring and heating at 55 °C in an Ar-filled glove box. Li_2Se_n solution was prepared by dissolving Li and Se with a molar ratio of 1:3 in DOL/DME mixed solution with volume ratio 1:1 and 5 vol% Li-SeS_2 electrolyte as additive, followed by magnetically stirring and heating at 55 °C in an Ar-filled glove box. Typically, the adsorption test was performed by adding the same amounts of CF, CF@CNTs, and CF@CNTs-NPP composites into 2 mL diluted Li_2S_n/Li_2Se_n solutions, respectively. As for the diffusion test, electrolyte and Li_2S_n/Li_2Se_n solutions were contained in a beaker and a vial with a holed-cap combined with an O-ring, respectively.

S1.2 Assembly of Symmetric Cells

Li_2S_6 catholyte was prepared by adding Li_2S and S with a molar ratio of 1:5 into Li-SeS_2 electrolyte contained 0.5 M LiTFSI and 1 wt% $LiNO_3$. Li_2S_6 catholyte was prepared by adding Li and Se with a molar ratio of 1:3 into Li–SeS_2 electrolyte contained 0.5 M LiTFSI and 1 wt% $LiNO_3$. CF, CF@CNTs, and CF@CNTs-NPP films with the diameters of 12 mm were used as both working and counter electrode, and 60 μL catholyte was added into each coin cell. The CV measurement of the symmetric cell was conducted at a scan rate of 1 mV s^{-1} with a voltage window between -1 and 1 V.

S1.3 Measurement for the Li_2S Nucleation

Nucleation of Li_2S on different electrodes were probed in 2032 coin cells with 30 μL 0.2M Li_2S_8 tetraglyme solution as catholyte and 15 μL of control electrolyte without Li_2S_8 was used as anolyte. Li foil was employed as the anode, CF, CF@CNTs, and CF@CNTs-NPP films with the diameters of 12 mm served as the working electrode. The assembled cells were first discharged galvanostatically at 0.112 mA to 2.06 V and then discharged potentiostatically at 2.05 V for Li_2S nucleation and growth until the current dropped below 10^{-5} A. It took about 25000 s and the energy was integrated to evaluate the capacities from deposition of lithium sulfide on various surfaces.

S1.4 Computational Details

The structure optimization was calculated with Gaussian software by the density functional theory of wB97XD/6-31G (*d*) and the results were visualized in VESTA. In order to simulate the interaction between Li_2S_n/Li_2Se_n and the pectic polysaccharides (NPP), four small molecule models, including galactose, glucose, galacturonic acid, and rhamnose, were built due to their similar functional groups with pectic polysaccharides. The binding energy (E_b) between Li_2S_6/Li_2Se_6 and NPP was defined as following:

$$E_b = E_{total} - E_{ads} - E_{LiPS} \qquad (S1)$$

Where E_{total} represents the total energy of NPP bound with Li_2S_6/Li_2Se_6, E_{ads}, E_{LiPS} are the isolated energies of the pristine NPP, and Li_2S_6/Li_2Se_6, respectively.

S1.5 Self-discharge Tests

The self-discharge rate (η%) was calculated by the following equations:

$$\eta\% = (C_{discharge(N-1)} - C_{discharge(N)})/C_{discharge(N-1)} \times 100\% \qquad (S2)$$

where $C_{discharge(N-1)}$ is the discharge capacity of the (N-l)th cycle, and $C_{discharge(N)}$ is the discharge capacity of the Nth cycle, respectively.

S2 Supplementary Figures

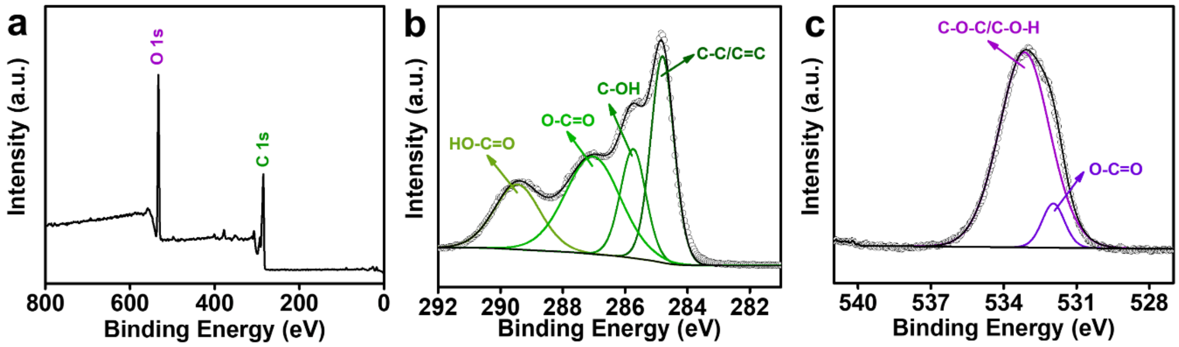

Fig. S1 XPS of NPP: (**a**) survey spectrum and high-resolution spectra of (**b**) C 1s and (**c**) O 1s

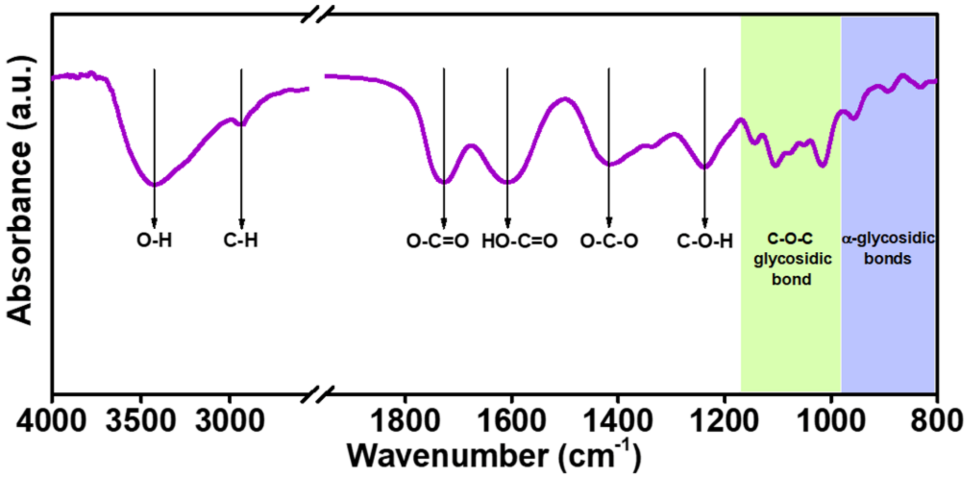

Fig. S2 FT-IR spectrum of NPP

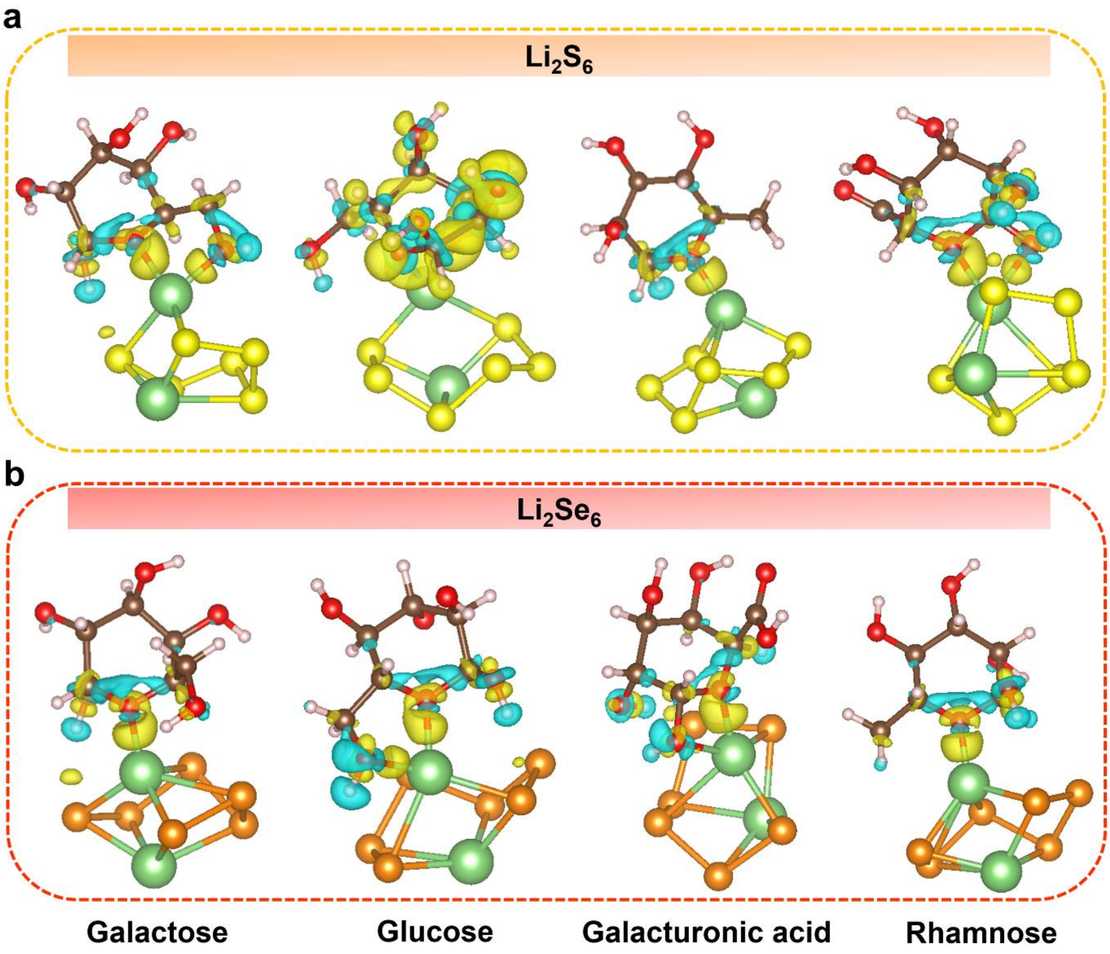

Fig. S3 Differential charge density of four typical constitutional units of NPP adsorbed with (**a**) Li_2S_6 and (**b**) Li_2Se_6. The selenium, sulfur, oxygen, lithium, carbon and hydrogen are distinguished with orange, yellow, red, green, brown and white. The yellow and blue surfaces correspond to the charge gain and lost regions, respectively (isovalue: 0.003).

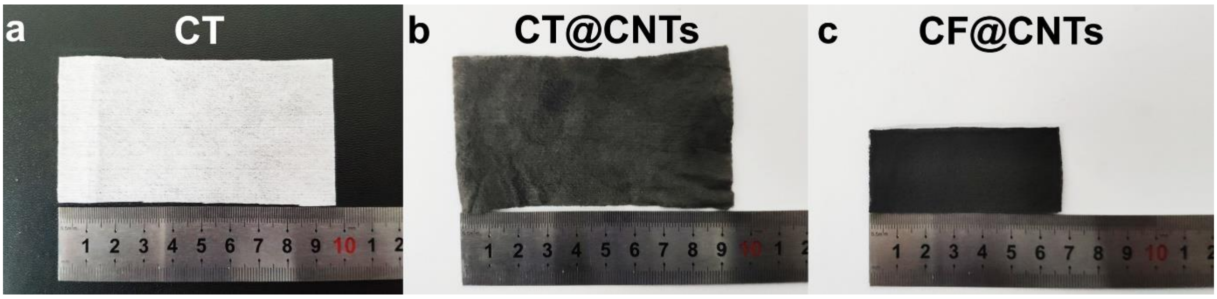

Fig. S4 Digital images of (**a**) CT, (**c**) CT@CNTs, and (**d**) CF@CNTs

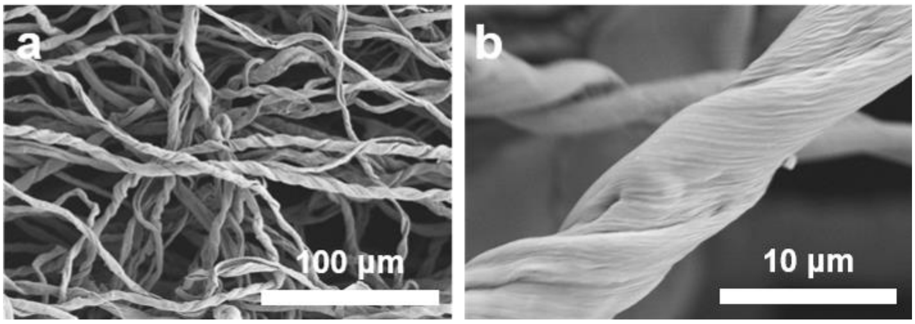

Fig. S5 Top-view FESEM images of CF

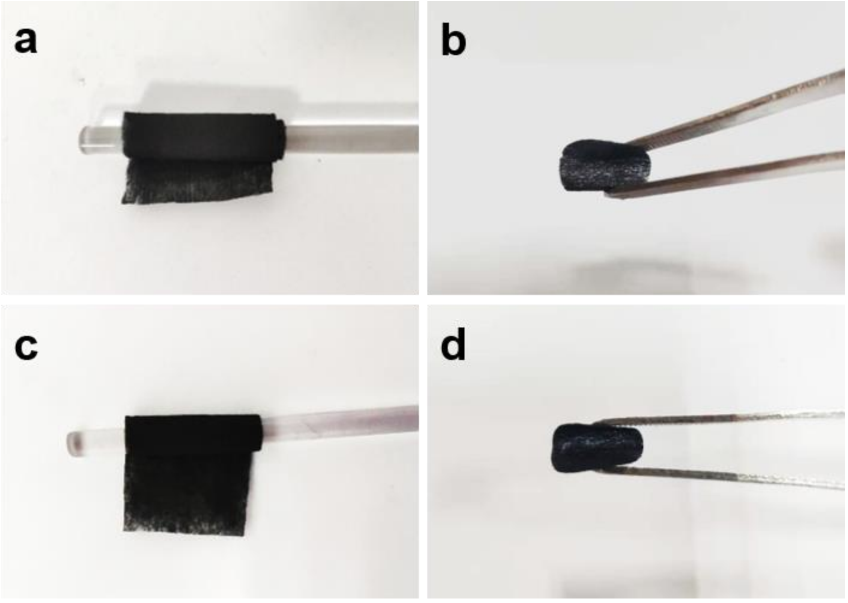

Fig. S6 Bending test on the (**a, b**) CF and (**c, d**) CF@CNTs interlayers

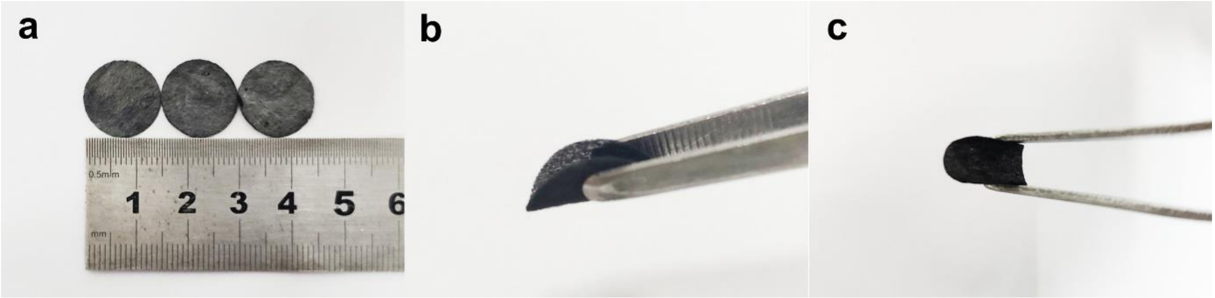

Fig. S7 Digital images of (**a**) the diameter length of three CF@CNTs-NPP and (**b**, **c**) bending test on the CF@CNTs-NPP interlayers

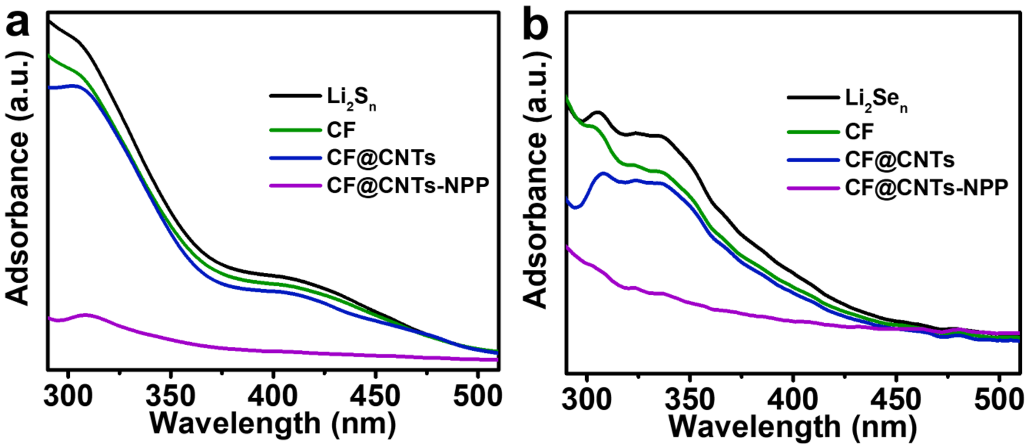

Fig. S8 UV-vis absorption spectra of (**a**) Li_2S_n and (**b**) Li_2Se_n solutions contacted with CF, CF@CNTs, and CF@CNTs-NPP after 12 h

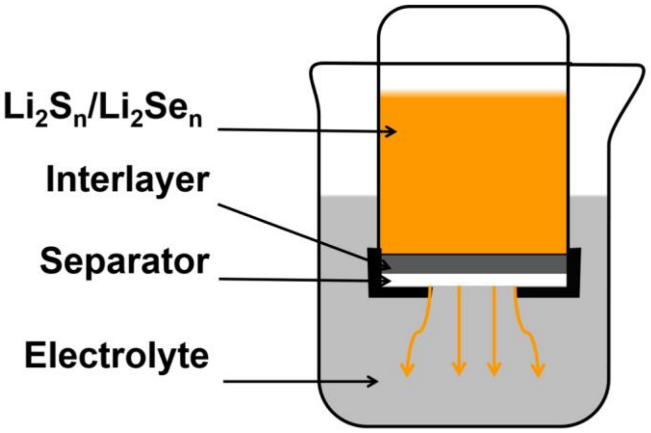

Fig. S9 Li_2S_n/Li_2Se_n permeation measurements: configuration of the diffusion test bottle showing the interlayer, separator, high concentrate Li_2S_n/Li_2Se_n solution, and blank electrolyte

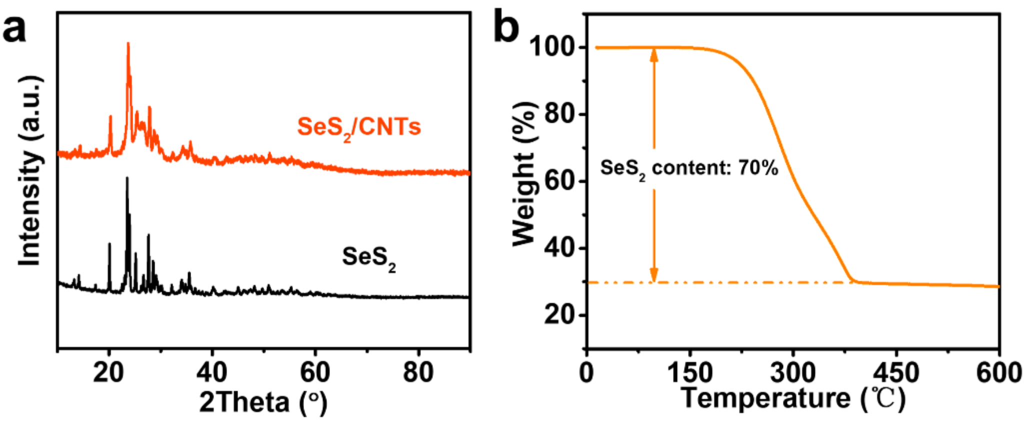

Fig. S10 (**a**) XRD patterns of SeS_2/CNTs and SeS_2. (**b**) TGA curve of SeS_2/CNTs in nitrogen atmosphere

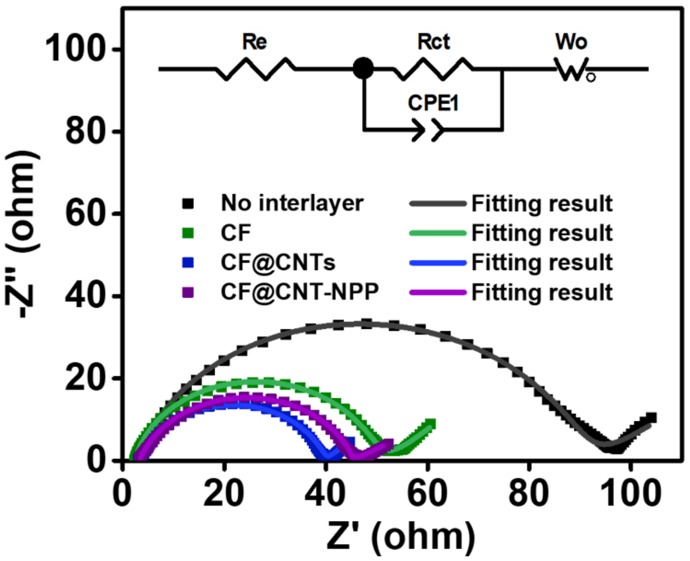

Fig. S11 Nyquist plots before cycling

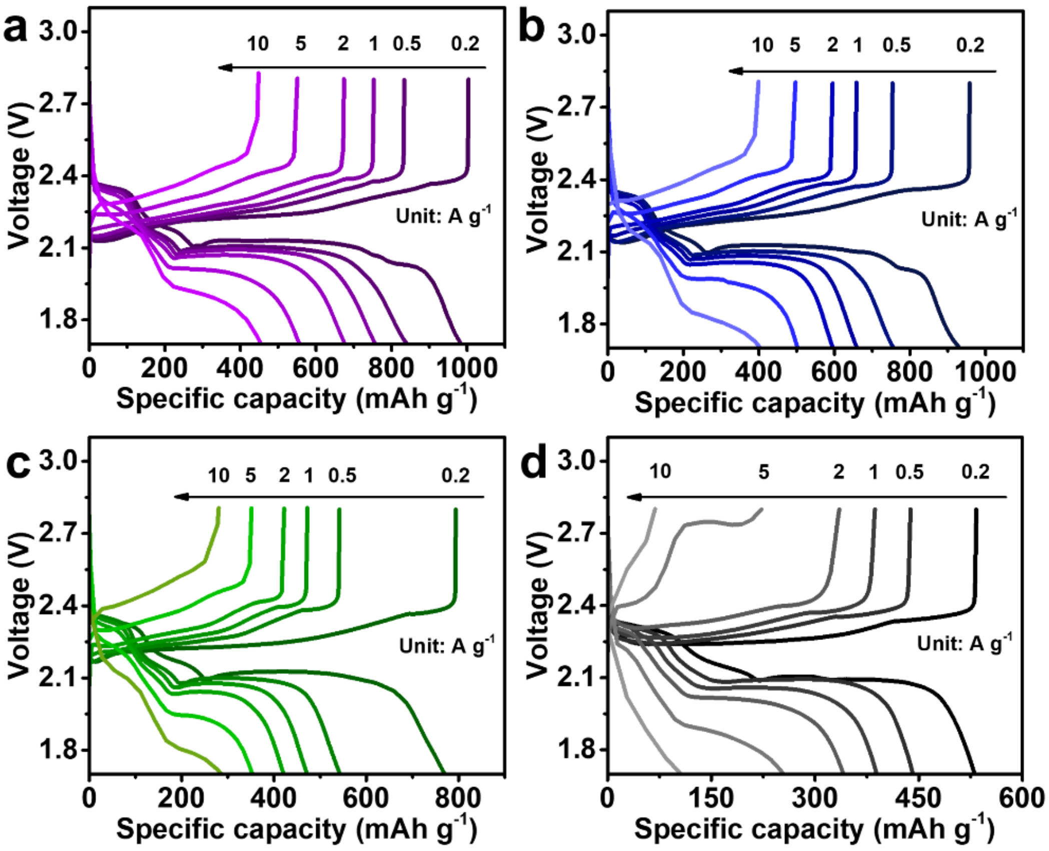

Fig. S12 Discharge-charge profiles of the cells with (**a**) CF@CNTs-NPP, (**b**) CF@CNTs, (**c**) CF interlayers, and (**d**) without interlayer at various current densities

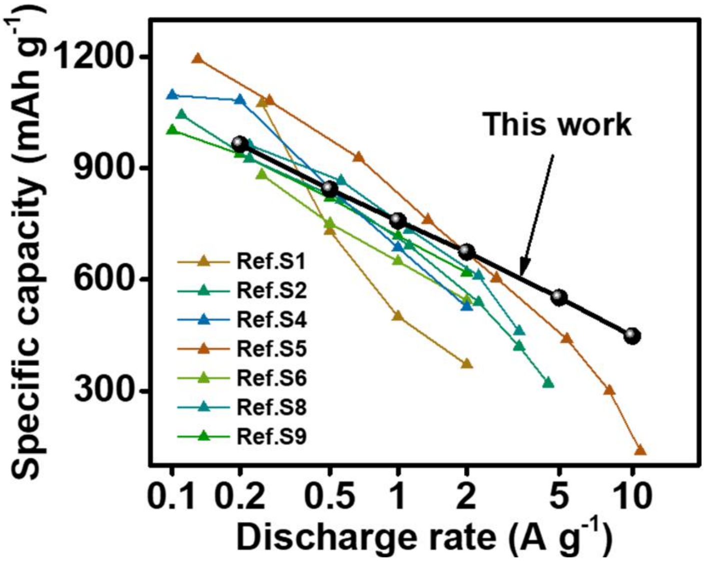

Fig. S13 Comparison of the rate capability with previously published Li-SeS_2 batteries

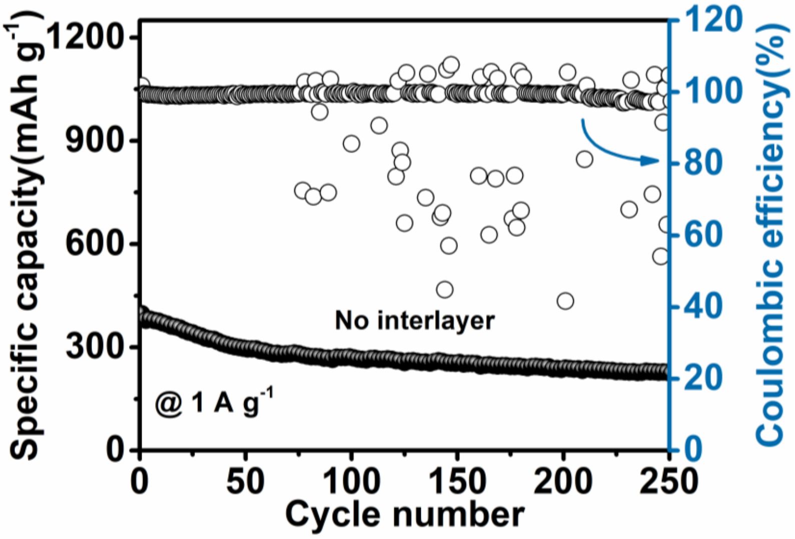

Fig. S14 Cycle performance at 1 A g^{-1} of the cell without interlayer

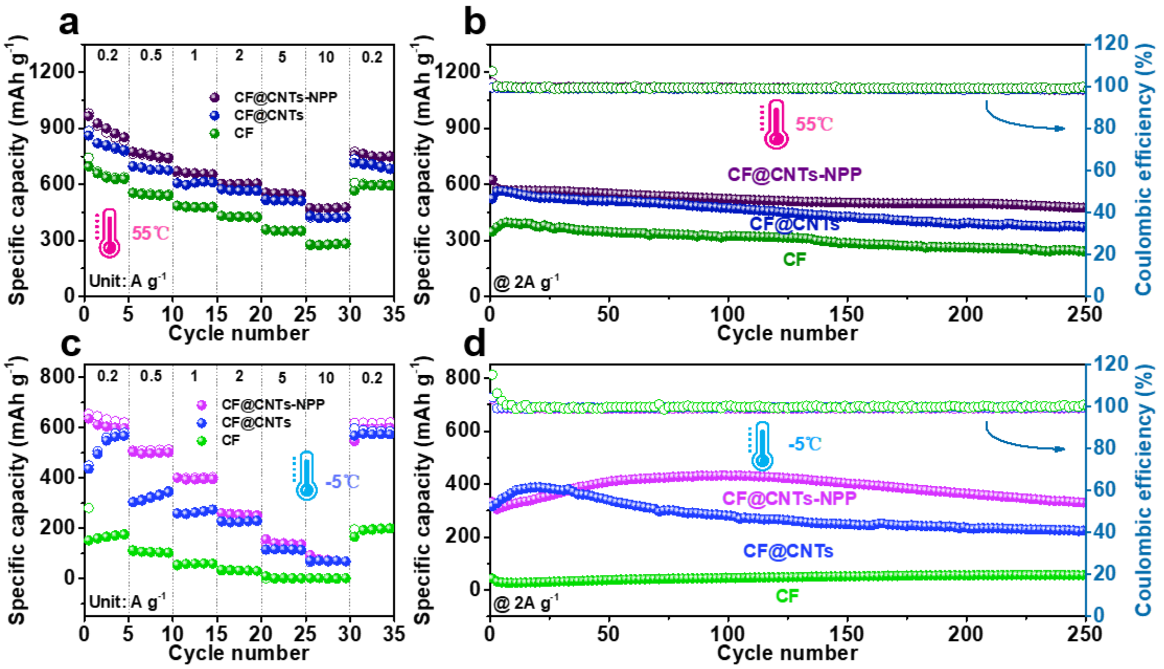

Fig. S15 Comparison of the rate capability and cycle performance under (**a**, **b**) raised and (**c**, **d**) subzero temperatures

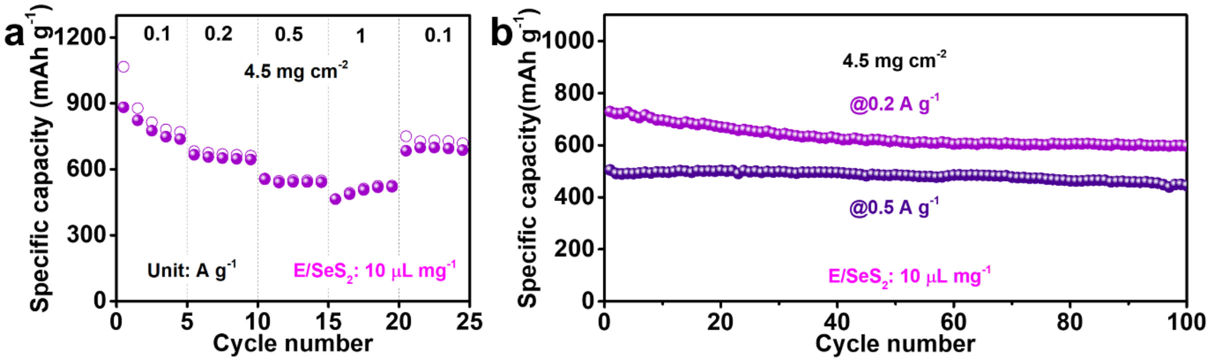

Fig. S16 Electrochemical performance under a SeS_2 areal loading of 4.5 mg cm^{-2} and a E/SeS_2 ratio of 10 μL mg^{-1}: (**a**) rate capability and (**b**) cycling performance at 0.2 A g^{-1} and 0.5 A g^{-1}

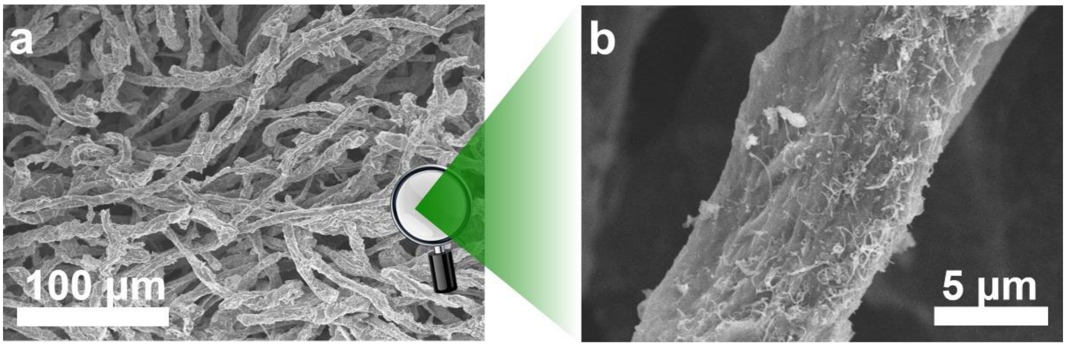

Fig. S17 FESEM images of the CF@CNTs-NPP interlayer toward the separator in Li–SeS_2 batteries after 100 cycles

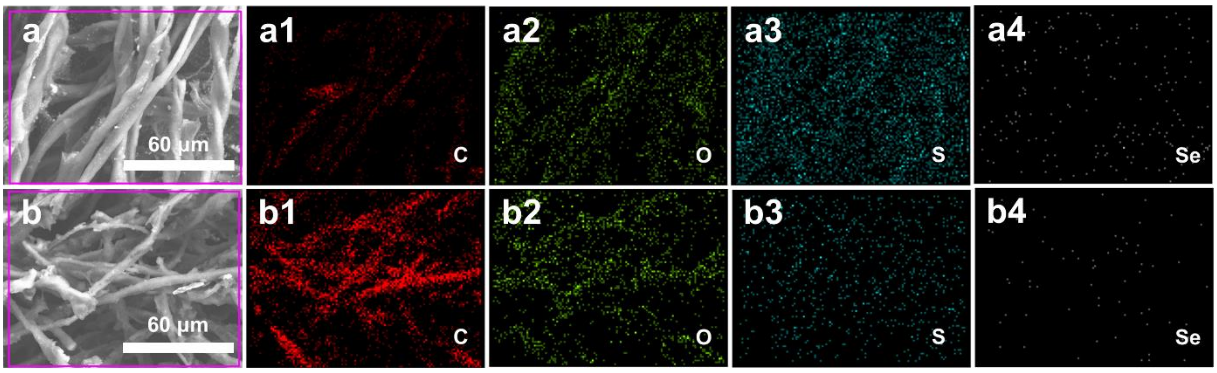

Fig. S18 Elemental mapping images of (**a**) CF@CNTs-NPP (the cathode side) and (**b**) CF@CNTs-NPP (the separator side) interlayer after 100 cycles

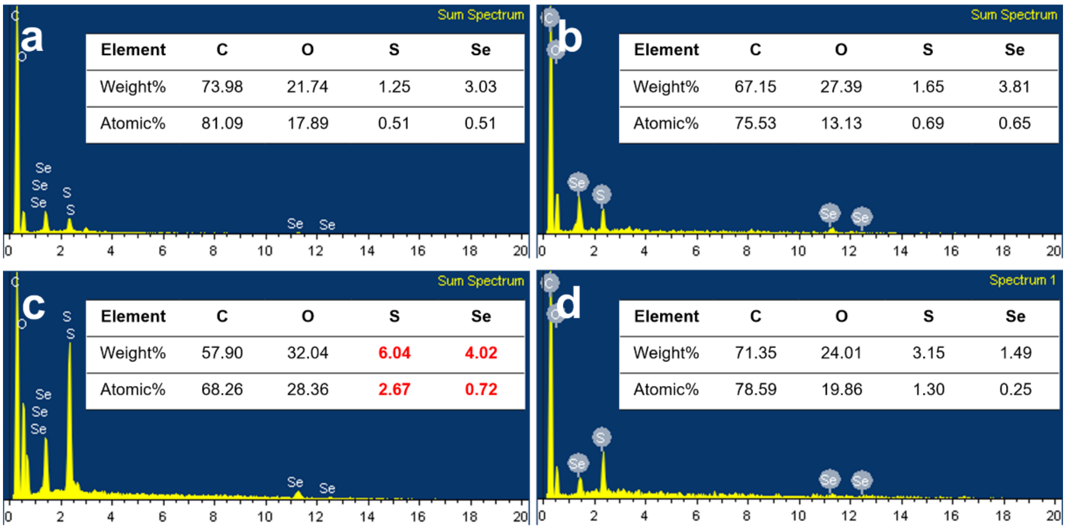

Fig. S19 EDX and component ratio of (**a**) CF, (**b**) CF@CNTs, (**c**) CF@CNTs-NPP (the cathode side) and (**d**) CF@CNTs-NPP (the separator side) interlayer after 100 cycles

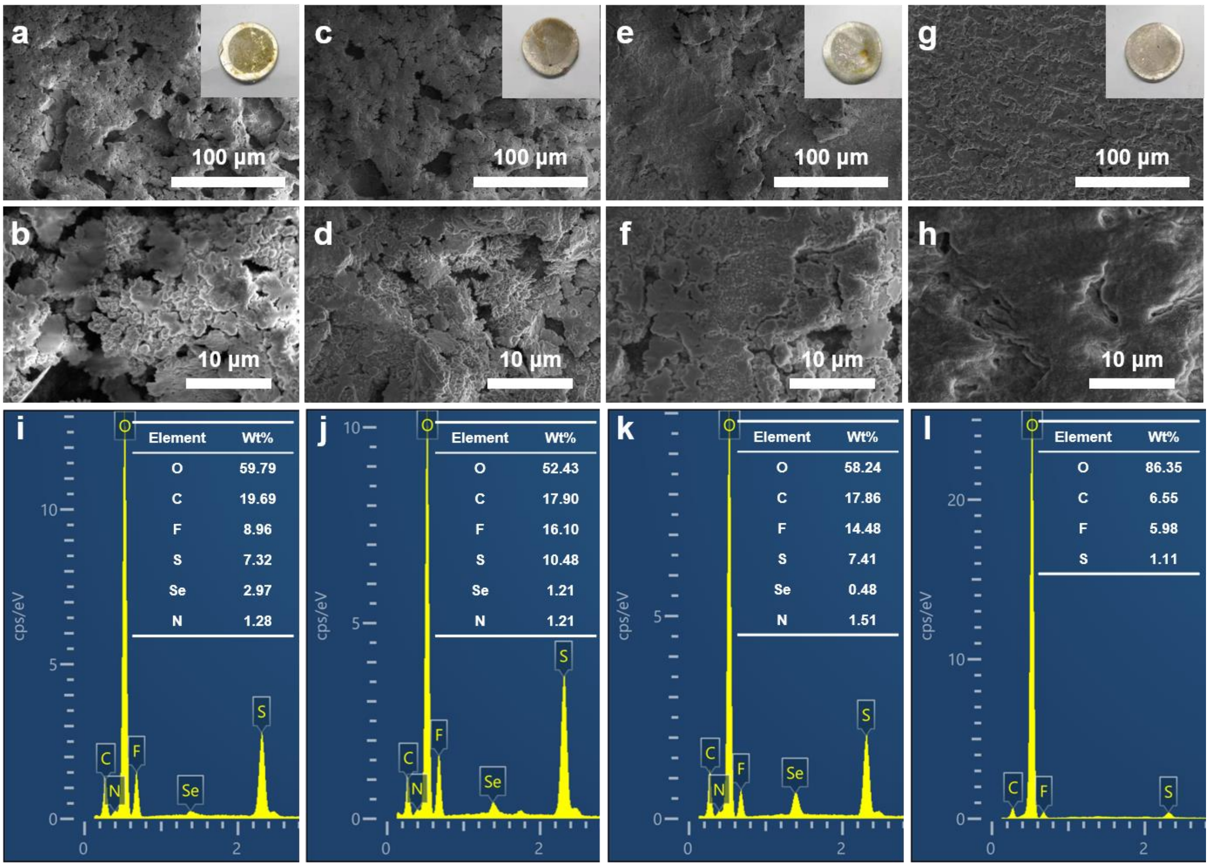

Fig. S20 FESEM images and optical photographs of lithium electrodes in the cells (**a, b**) without interlayer and with (**c, d**) CF, (**e, f**) CF@CNTs, and (**g, h**) CF@CNTs-NPP interlayers after 10 cycles at 1 A g^{-1}. (**i-l**) The corresponding EDX and component ratio

S3 Supplementary Tables

Table S1 Square resistance values of the samples

Samples	CF	CF@CNTs	CF@CNTs-NPP
Resistance (Ω/□)	28.97	11.10	13.08

Table S2 Comparison of electrochemical performance with previously reported SeS_2-based cathodes in Li-SeS_2 batteries

Sample	Content of SeS_2 (wt.% / mg cm^{-2})	Rate capability (mAh g^{-1})	Cycle performance (mAh g^{-1})			Refs.
		Capacity/rate	Rate/cycle numbers	Initial capacity	Decay rate (per cycle)	
SeS_2/MCA	49.3/1.5-2	371/2 A g^{-1}	0.5 A g^{-1}/250	846	NA	[S1]
HMC/TiN/ SeS_2	70/1	320/4 C	0.2 C/200	987	0.15%	[S2]
CMK-3/ SeS_2@PDA	70/2.6-3	535/5 A g^{-1}	2 A g^{-1}/500	866	0.11%	[S3]
CoS_2 @LRC/SeS_2	70/2.3-3	526/2 A g^{-1}	0.5 A g^{-1}/400	868	0.10%	[S4]
Co-N-C/ SeS_2	66.5/3.2	138.1/8 C	0.2 C/200	1153.5	0.08%	[S5]

SeS_2/ISMC	49.7/1	465/4 A g^{-1}	0.5 A g^{-1}/200	858	0.31%	[S6]
$NiCo_2S_4$@NC -SeS_2	66.7/2	673.5/2 A g^{-1}	1 A g^{-1}/800	678.6	0.038%	[S7]
MYS-Co_4N @C/SeS_2	70/1.2	460/3 C	0.5 C/300	909	0.088%	[S8]
PANI-DSMC-SeS_2	70/1.6-2.0	619/2 A g^{-1}	2 A g^{-1}/500	~700	~0.094%	[S9]
**This work**	**70/1.3**	**448/10 A g^{-1}**	**2 A g^{-1}/800**	**671**	**0.037%**	

Supplementary References

[S1] Z. Zhang, S. Jiang, Y. Lai, J. Li, J. Song et al., Selenium sulfide@mesoporous carbon aerogel composite for rechargeable lithium batteries with good electrochemical performance. J. Power Sources **284**, 95-102 (2015). https://doi.org/10.1016/j.jpowsour.2015.03.019

[S2] Z. Li, J. Zhang, B. Y. Guan, X. W. D. Lou, Mesoporous carbon@titanium nitride hollow spheres as an efficient SeS_2 host for advanced Li-SeS_2 batteries. Angew. Chem. Int. Ed. **56**(50), 16003-16007 (2017). https://doi.org/10.1002/anie.201709176

[S3] Z. Li, J. Zhang, H. B. Wu, X. W. D. Lou, An improved Li-SeS_2 battery with high energy density and long cycle life. Adv. Energy Mater. **7**(15), 1700281 (2017). https://doi.org/10.1002/aenm.201700281

[S4] J. Zhang, Z. Li, X. W. D. Lou, A freestanding selenium disulfide cathode based on cobalt disulfide-decorated multichannel carbon fibers with enhanced lithium storage performance. Angew. Chem. Int. Ed. **56**(45), 14107-14112 (2017). https://doi.org/10.1002/anie.201708105

[S5] J. He, W. Lv, Y. Chen, J. Xiong, K. Wen et al., Direct impregnation of SeS_2 into a MOF-derived 3D nanoporous Co–N–C architecture towards superior rechargeable lithium batteries. J. Mater. Chem. A **6**(22), 10466-10473 (2018). https://doi.org/10.1039/c8ta02434k

[S6] J. Hu, H. Zhong, X. Yan, L. Zhang, Confining selenium disulfide in 3D sulfur-doped mesoporous carbon for rechargeable lithium batteries. Appl. Surf. Sci. **457**, 705-711 (2018). https://doi.org/10.1016/j.apsusc.2018.06.296

[S7] B. Guo, T. Yang, W. Du, Q. Ma, L.-Z. Zhang, S.-J. Bao et al., Double-walled N-doped carbon@$NiCo_2S_4$ hollow capsules as SeS_2 hosts for advanced Li–SeS_2 batteries. J. Mater. Chem. A **7**(19), 12276-12282 (2019). https://doi.org/10.1039/c9ta02695a

[S8] T. Chen, W. Kong, M. Fan, Z. Zhang, L. Wang et al., Chelation-assisted formation of multi-yolk–shell Co_4N@carbon nanoboxes for self-discharge-suppressed high-performance Li–SeS_2 batteries. J. Mater. Chem. A **7**(35), 20302-20309 (2019). https://doi.org/10.1039/c9ta07127j

[S9] J. Hu, Y. Ren, L. Zhang, Dual-confined SeS_2 cathode based on polyaniline-assisted double-layered micro/mesoporous carbon spheres for advanced Li–SeS_2 battery. J. Power Sources **455**, 227955 (2020). https://doi.org/10.1016/j.jpowsour.2020.227955
